# Single nucleotide polymorphisms in candidate genes are significantly associated with resistance to *Haemonchus contortus* infection in goats

**DOI:** 10.1186/s40104-019-0327-8

**Published:** 2019-03-15

**Authors:** Mahmuda Bilkis Bintee Alam, Abdullah Ibne Omar, Md. Omar Faruque, David Russell Notter, Kathiravan Periasamy, Md. Motahar Hussain Mondal, Md. Jalal Uddin Sarder, Md. Shamsuddin, Jianhua Cao, Xiaoyong Du, Zhenyang Wu, Shuhong Zhao

**Affiliations:** 1 Key Lab of Agricultural Animal Genetics, Breeding and Reproduction, Ministry of Education, College of Animal Science and Technology, Huazhong Agricultural University, Wuhan, 430070 People’s Republic of China; 2 National Engineering Laboratory for Animal Breeding, Key Lab of Agricultural Animal Genetics, Breeding and Reproduction, Ministry of Agriculture, College of Animal Science and Technology, China Agricultural University, Beijing, 100193 People’s Republic of China; 3 Department of Animal Breeding and Genetics, Bangladesh Agricultural University, Mymensingh, 2202 Bangladesh; 4 Department of Animal and Poultry Sciences, Virginia Tech, Blacksburg, VA 24061 USA; 5 Animal Production and Health Laboratory, Join FAO/IAEA Division of Nuclear Techniques in Food and Agriculture, International Atomic Energy Agency, Vienna, Austria; 6 Department of Parasitology, Bangladesh Agricultural University, Mymensingh, 2202 Bangladesh; 7 Department of Veterinary and Animal Science, University of Rajshahi, Rajshahi, 6205 Bangladesh; 8 Agricultural Bioinformatics Key Laboratory of Hubei Province, College of Informatics, Huazhong Agricultural University, Wuhan, 430070 People’s Republic of China; 9 College of Agroforestry Engineering and Planning, Tongren Univesity, Tongren, Guizhou 554300 People’s Republic of China

**Keywords:** Fecal egg count, Goat, *Haemonchus contortus*, Parasite resistance, SNP markers

## Abstract

**Background:**

Haemonchosis is a major economic problem in goat production in humid, tropical and subtropical regions. The disease is caused by an abomasal nematode, *Haemonchus contortus*, which is highly pathogenic in small ruminants*.* The aim of this study was to identifying single-nucleotide polymorphisms (SNP) that were associated with fecal egg counts (FEC) and could be used as markers to identify resistance to *H. contortus* in goats.

**Results:**

Ten novel variants in the *CIITA, ATP2A3, HSPA8, STAT5B, ESYT1*, and *SERPING1* genes were associated with FEC in goats with a nominal significance level of *P* < 0.05. Two missense mutation in the exon region of the caprine *CIITA* gene resulted in replacement of arginine with cysteine at position 9473550 (R9473550C) and aspartic acid with glutamic acid at position 9473870 (D9473870E). Chinese goat breeds had significantly higher FEC than Bangladeshi goat breeds within their respective genotypes. Polymorphism information content (*PIC*), effective allele number (*Ne*), and heterozygosity (*He*) were greatest for the *STAT5B*_197_A > G SNP locus in all goat breeds. Pairwise coefficients of linkage disequilibrium (D´, *r*^2^) revealed complete LD (*r*^2^ = 1) between significant SNP polymorphisms in *CIITA* and *SERPING1* and strong LD (*r*^2^ = 0.93 and 0.98) between polymorphisms in *HSPA8* and *ATP2A3*, respectively. Correlation coefficient (*r*) between FEC and body weight (BW) was significantly positive (*r* = 0.56***, *P* < 0.001) but that between FEC and packed cell volume (PCV) was negatively significant (*r* = − 0.47**, *P* < 0.01) in the total population of goats. On the other hand, correlation coefficient (*r*) between BW and PCV was not significant in total population of goats. Association analysis revealed that haplotypes within *ATP2A3, HSPA8*, and *SERPING1* were significantly associated with FEC. Quantitative real-time PCR revealed that the relative expression of mRNA was higher (*P* < 0.001) for resistant, compared to susceptible, groups of goats for all candidate genes except *CIITA*.

**Conclusions:**

This study identified SNP markers that can potentially be used in marker-assisted selection programs to develop goat breeds that are resistant to *H. contortus.*

**Electronic supplementary material:**

The online version of this article (10.1186/s40104-019-0327-8) contains supplementary material, which is available to authorized users.

## Background

Livestock, and especially small ruminants, provide one of the principal means to improve living standards in many developing countries and play a critical role in both the national economy and livelihoods in rural communities [1, 2]. Among small ruminant species, goats are particularly susceptible to gastrointestinal nematode (GIN) parasites in humid, tropical and subtropical regions where goats are reared under natural grazing conditions and are especially affected by *Haemonchus contortus* (*H. contortus*), haematophagous abomasal nematode parasite [3, 4]. Haemonchosis causes significant economic losses due to negative effects on production and increases in the cost of anthelmintic treatment [5–7].

Livestock producers and breeders are therefore trying to utilize genetic variation in susceptibility to parasitic infection within and among breeds to develop lines that are resistant to *H. contortus* [8–10]. The advance of molecular genetics has provided an opportunity to identify genes and associated pathways which provide a better understanding of the physiological processes associated with targeted genes and can potentially be used as genetic markers in marker-assisted selection programs [11]. Several candidate genes associated with parasite susceptibility have been identified and partially characterized in different farm animal species. However, only a few studies have addressed candidate genes and polymorphisms associated with resistance to GIN in goat [6, 12, 13].

Gene expression studies revealed that resistant animals expressed hundreds of genes involved in immune function [14]. Genetic variants that affect phenotypic variation are generally identified by association analysis [15]. Such association studies have already been done in different animals including human [16, 17]. Major histocompatibility complex class II molecules (*CIITA*), the endoplasmic Ca^2+^ pump (*ATP2A*3), heat stress protein 70 (*HSPA8*), serpin peptidase inhibitor clade G member 1 (*SERPING1*), signal transducer activator of transcription 5B (*STAT5B*), and extended synaptotagmin-1 (*ESYT1)* have been shown to impact immune function in humans and several livestock species. Polymorphisms in these genes have been shown to be associated with autoimmune diseases in human [18], mice [17], and other farm animal species such as sheep, goat, yak, buffalo, and chicken [16, 19, 20], but comparable association studies involving resistance to GIN and, particularly, to *Haemonchus contortus* in goats have not been reported. The present research was therefore undertaken to search for associations between single-nucleotide polymorphism (SNP) in *CIITA, ATP2A3, HSPA8, STAT5B, ESYT1* and *SERPING1* and resistance to *H. contortus* using fecal egg counts (FEC) as an indicator of the level of parasite infection.

## Methods

### Ethics statement

All experiment were carried out in accordance with the Guide for the Care and Use of Laboratory Animals (Ministry of Science and Technology of China, 2006) and approved by the Standing Committee of the Hubei People’s Congress, and the Ethics Committee of Huazhong Agricultural University, Wuhan, China (Permission Number 4200896859).

### Experimental animals

A total of 507 goats were used for the study and included 32 Yichang White (YCW), 56 Nanjiang Yellow (NJY), 37 Enshi Black (ESB), and 155 crossbred goats (CCB; produced by the mating YCW, NJY, and ESB goats) from China and 227 Black Bengal goats (BBG) from Bangladesh. Experimental animals were all grazed on native pastures and had not been dewormed for at least 1 year to maximize the likelihood of natural parasite infection. Goats were selected from three locations: Enshi city (30^o^ N, 109^o^ E) and Yichang city (31^o^ N, 111^o^ E) in Hubei province in southern China and the Natore district (24^o^ N, 89^o^ E) in western Bangladesh. The animal pedigree was ensured from the record of animals maintained by the farmers as well as experimental record on the animals, especially in Bangladesh.

### Phenotypic data and sample collection

The data were collected at the end of rainy season, from June to August 2015, when parasitic infection was maximum due to high moisture levels and ambient temperatures [3]. Body weights were determined and fecal and peripheral blood samples were collected from each goat. The FEC was determined using a modified McMaster’s technique [21, 22]. Blood was collected using venoject tubes coated with ethylene diaminetetraacetic acid (EDTA) as an anti-coagulant and transferred to a laboratory for DNA extraction using the standard phenol–chloroform method [23] and determination of packed cell volume (PCV) using the capillary micro-hematocrit method [24].

### Targeted re-sequencing and identification of polymorphisms

A total of 84 candidate genes were identified for targeted re-sequencing and overall 129 polymorphisms (the number of SNP ranged from 1 to 6 for each of the genes) were identified in goats that were involved in immune system and body defense mechanisms. The candidate genes (NCBI Gen Bank association numbers presented in Additional file 1: Table S1) were selected based on analysis of the global list of sheep Entrez Gene IDs in the bovine KEGG database using KEGGARRAY. For the amplification of polymerase chain reaction (PCR) product, oligonucleotide primers were designed for partial regions of genes in a panel of eight unrelated goats selected from different breeds located in major environmental areas under present study. The final PCR reaction volume was 50 μL consisting of a 2-μL template containing 50 ng of genomic DNA, 1.5 μL of each primer, 20 μL of double-distilled H_2_O, and 25 μL of Taq premix (TaKaRa, Dalian. China). For all primers, samples were incubated at 94 °C for 10 min and then amplified for 35 cycles; each cycle consisted of denaturation at 95 °C for 30 s, annealing at 58 °C for 30 s, an extension at 72 °C for the 50 s, and the final extension at 72 °C for 5 min. The PCR products were checked by electrophoresis on 1% agarose gel to confirm amplification and sequenced using an ABI 3730XL genetic analyzer (Tsingke Biological Technology Co. Ltd., Wuhan, China). Sequences were generated from both ends, and single nucleotide polymorphisms were identified using Codon Code Aligner version 8.0.1 and BioEdit software [25] and confirmed by manual inspection.

### SNP genotyping

After identification of the candidate SNP, the animals were genotyped using a Kompetitive allele-specific PCR (KASPar) assay based on FRET chemistry (KBiosciences, LGC Genomics. UK). Two forward primers specific to each SNP allele were designed and combined with the respective proprietary tail sequence complementing the FAM or HEX fluorescence reporting system [26]. A common reveres primer was designed for each genotyping assay. Thermal cycling conditions are presented in Additional file 1: Table S2. Genotype calling was based on an endpoint allele discrimination module to measure fluorescence intensities recorded for each of two alleles. The emission data for all the samples on the plate were plotted on corresponding *X* and *Y* axis for each allele, and the genotypes were called based on distinct clustering [26]. Touchdown PCR (TD-PCR) assays were used to amplify the variable regions of the different DNA sequences and allow genotyping of each of the target SNP locus. The TD-PCR protocol used a total of 36 cycles of amplification, each including two steps of extension and annealing processes. Touchdown PCR protocol (61–55 TD) was used for genotyping of *CIITA* and *ATP2A3* and protocol (68–62 TD) was used for genotyping of *HSPA8, STAT5B, ESYT1* and *SERPING1* (Additional file 1: Table S2).

### Artificial challenge trial

An artificial challenge with *H. contortus* was performed at the Lao Gao Huang goat farm, Yichang, China to asses mRNA expression for each candidate gene in goats characterized as resistant or susceptible to *H. contortus*. An initial sample of 81 Yichang White goats of both sexes with ages of 9 to 12 months was selected from a herd of 450 goats and FEC, circulating haemoglobin levels (Hg), and PCV were determined for those animals. Goats were then divided into two groups based on their FEC. Goats with low FEC (FEC < 500**)** were assigned to the resistant group and goats with high FEC (FEC > 500) were assigned to the susceptible group. From these groups, 4 resistant goats and 4 susceptible goats with an average age of 305 ± 10 days were selected for artificial challenge. Experimental goats were moved to drylot, treated with Ivermectin (0.25 mg/kg body weight), and, 15 days later, with levamisole (8 mg/kg body weight) [27, 28]. In dry lot, goats were fed GIN-free fresh grass, and a concentrate mixture was provided twice per day at a rate of 1.5% of body weight; pens was cleaned regularly. Complete deworming was confirmed by determination of FEC. When all experimental goats achieved a FEC of zero, resistant and susceptible goats were inoculated with a single dose of 5000 third-stage (L3) larvae of *H. contortus*. The day of inoculation was considered to be day 0, and FEC, PCV, Hg, and body weight (BW) were determined weekly for 6 weeks after inoculation. At day 42, all experimental goats were sacrificed, contents of the abomasum and small intestine were collected, and mucosa was thoroughly washed into the same containers. Worms from the abomasum and small intestine were then counted.

### RNA extraction, cDNA synthesis and qRT-PCR

Approximately 100 g of abomasal tissue including lymph node from the hemorrhagic part of the abomasal wall were collected and total RNA was extracted using TRlzol (Invitrogen, Carlsbad, CA, USA) following the manufacturer’s recommended procedure. A Nano Drop ND2000 spectrophotometer (Thermo Fisher Scientific, Waltham, MA, USA) was used to assess the quality of extracted RNA that was utilized to mRNA expression for *ATP2A3, SERPING1, CIITA, HSPA8, ESYT1*, and *STAT5B* by quantitative real-time PCR (qRT-PCR) using cDNA generated from approximately 1 μg of RNA using a standard PrimeScipt™ RT reagent kit with gDNA eraser (Perfect Real Time, TAKARA Bio, Inc.). The qRT-PCR was performed using a CFX-96 Bio-Rad thermal cycler with SYBR green real-time PCR master mix (Toyobo Co., Ltd., Osaka, Japan). The qRT-PCR protocol was a single cycle of denaturation at 95^°^*C* for 5 min, 45 cycles of denaturation at 95 °C for 20 s, annealing at 58 °C for 20 s, and extension at 72 °C for 15 s*. β*-actin (*ACTB*) was used as a housekeeping gene to normalize the samples. Primers used for qRT-PCR are shown in Additional file 1: Table S3.

### Statistical analysis

#### Descriptive statistics

The FEC were not normally distributed and exhibited positive skewness. A logarithmic transformation [log_10_ (FEC + 25)] was therefore applied before analysis [9, 29]. Descriptive statistics (arithmetic means, variances, standard errors, etc.; [30]) were derived using SAS (Version 9; SAS Institute, Inc., Cary, NC USA). No transformation was applied to PCV or body weight.

#### Polymorphism evaluation

Genotypic and allelic frequencies were determined for each candidate genes in each population [31]. Correspondence of observed genotypic frequencies to expectations based on Hardy-Weinberg equilibrium (HWE) was assessed by chi-square (*χ*^2^) tests in POPGENE software (Version 3.2;) [32]. Population genetic diversity indices [gene heterozygosity (*He*), polymorphism information content (*PIC*), and effective allele numbers (*Ne*)] were calculated using PICcalculator an online program available at https://www.liverpool.ac.uk/~kempsj/pic.html [33–35]. Heploview blocks and linkage disequilibrium (LD) measures (D′, *r*^2^) were derived for the significant SNP loci using genotypes for all goats in this study and the default settings in the Heploview software. Descriptive statistics were estimated using the partition–ligation, combination–subdivision expectation maximization algorithm [36] in Heploview software [11, 37].

#### Association analysis

Relationships between SNP and FEC were tested using R software [38] and a linear model:

$$ \boldsymbol{y}=\boldsymbol{\mu} +\boldsymbol{B}+\boldsymbol{V}+\boldsymbol{G}+\boldsymbol{e} $$

where ***y*** is the FEC, ***μ*** is the overall population mean, ***B*** and ***V*** are fixed effects of breed and location associated with the FEC trait, ***G*** is one of the 129 SNP which were tested for an association with FEC, and ***e*** is residual error [39]. Multiple comparisons between genotypes of significant SNP and fecal egg count (FEC) in all goat population were carried out using R software (Version 3.0.2).

#### Evaluation of gene expression in the artificial challenge trial

Differences in gene expression between resistant and susceptible goats in the challenge trial were tested using the 2^-ΔΔCT^ method in SAS [40, 41]. Student’s *t*-tests and a significance level of *P* < 0.05 were used to compare gene expression in resistant and susceptible goats using Graph Pad Software Prism7 (San Diego, CA USA).

## Results

### Descriptive statistics

Based on FEC, 237 out of 507 goats were infected with *H. contortus*, resulting in an overall infection rate as 46.75%. Average infection rates were 68.9% for Chinese goat breeds and 19.4% for BBG goats. Least-squares means for FEC and PCV differed (*P* < 0.001) between goats from China and Bangladesh. Least-squares means for FEC and PCV also differed (*P* < 0.001 and *P* < 0.05, respectively) among breeds, but not between locations, in China (Table 1). Across the five goat breeds evaluated in this study, transformed FEC were highest (2.53 ± 0.10 epg) for ESB goats and lowest (1.58 ± 0.04 epg) for BBG goats. However, the maximum FEC (15,600 epg) was observed in a Chinese crossbred goat (Table 1). Male goats had higher FEC than female goats (*P* < 0.01), but did not differ from female goats in PCV; this result was consistent across countries In contrast to results for FEC, body weights were similar among breeds, locations, and sexes (Table 1).

**Table 1:** Least-square means and standard errors for log-transformed fecal egg count, body weight, and packed cell volume for goats exposed to natural infection with *H. contortus* in China and Bangladesh

Main effect	Class	No.	LFEC (log_10_)		Body weight, kg		PCV, %	
			Mean ± SE	Max.	Mean ± SE	Range	Mean ± SE	Range
China								
Location	Yichang	182	2.34 ± 0.05^a^	15,600	26.13 ± 0.2.3	6 to 33	17.08 ± 0.48	17 to 35
	Enshi	98	2.45 ± 0.06^b^	9400	25.83 ± 0.32	8 to 36	17.68 ± 0.66	17 to 33
			ns		ns		ns	
Breed	YCW	32	2.25 ± 0.11^a^	2400	26.15 ± 0.57	6 to 28	18.72 ± 0.98^b^	18 to 33
	NJY	56	2.08 ± 0.08^b^	5400	25.92 ± 0.42	6 to 32	17.96 ± 0.86^c^	17 to 32
	ESB	37	2.53 ± 0.10^a^	3600	26.27 ± 0.52	9 to 36	20.35 ± 0.99^a^	18 to 33
	Chinese crossbred	155	2.48 ± 0.05^c^	15,600	25.83 ± 0.25	6 to 33	17.99 ± 0.52^c^	18 to 35
			***		ns		*	
Bangladesh								
Breed	BBG	227	1.58 ± 0.04	400	26.53 ± 0.21	6 to 35	20.16 ± 0.43	18 to 35
China & Bangladesh								
Sex	Male	151	2.09 ± 0.04^a^	15,600	26.30 ± 0.17	6 to 36	17.55 ± 0.37	17 to 35
	Female	356	1.86 ± 0.06^b^	7200	26.14 ± 0.26	6 to 35	17.39 ± 0.56	17 to 32
			**		ns		ns	

Goats from China and Bangladesh differed (*P* < 0.001) for LFEC and PCV but not body weight

Fecal egg counts (FEC) were transformed as LFEC = log_10_ [(FEC + 25)]

*SE* standard error, *PCV* packed cell volume, *YCW* Yichang White, *NJY* Nanjiang Yellow, *ESB* Enshi Black, *BBG* Black Bengal Goat

Significance levels for each main effect and measured variable are shown on the line following the last main effect class. ***: *P* < 0.001; **: *P* < 0.01; ns: not significant

^a, b, c^Means within a country designation and main effect with different superscripts differ (*P* < 0.05)

### Identification of polymorphisms

Out of 129 polymorphisms from 84 different genes, 10 polymorphisms in 6 genes (*CIITA, ATP2A3, HSPA8, STAT5B, ESYT1* and *SERPING1*) had significant associations with FEC at a nominal significance level of *P* < 0.05. The rest of polymorphisms (119 polymorphisms in 78 genes) had non-significant associations with the studied phenotype (FEC) are presented in Additional file 1: Table S5. Two non-synonymous mutations in *CIITA* resulted in replacement of arginine with cysteine at position 9473550 and aspartic acid with glutamic acid at position 9473870 (Table 2). The remaining eight SNP were located in non-coding regions and included a SNP in the 3´UTR of *STAT5B* (A/G at position 41961106), two SNP in the 3´UTR of *ATP2A3* (A/G at position 24358932 and A/C at position 24358402), and five SNP in various intron regions (Table 2).

**Table 2:** Candidate genes and descriptions of SNP

Gene	SNP	Nucleotide		Chromosome No.	Amino acid	
		Position	Alleles		Encode	Region
*CIITA*	*CIITA*_161_C > T	9473550	C/T	25	R > C	Coding
*CIITA*	*CIITA*_481_A > T	9473870	A/T	25	D > E	Coding
*HSPA8*	*HSPA8*_1024_A > G	48655334	A/G	15	–	Intron8–9
*HSPA8*	*HSPA8*_1064_A > G	48655374	A/G	15	–	Intron8–9
*ESYT1*	*ESYT1*_559_G > C	56567955	G/C	5	–	Intron5–6
*STAT5B*	*STAT5B*_197_A > G	41961106	A/G	19	–	3’UTR
*SERPING1*	*SERPING1*_312_C > T	2421828	C/T	15	–	Intron6–7
*SERPING1*	*SERPING1*_615_G > T	2422132	G/T	15	–	Intron5–6
*ATP2A3*	*ATP2A3*_150_A > G	24358932	A/G	19	–	3’UTR
*ATP2A3*	*ATP2A3*_680_A > C	24358402	A/C	19	–	3’UTR

Amino acid symbols: R = Arginine, C = Cysteine, D = Aspartic acid, E = Glutamic acid

### Genotypic and allelic frequencies

Across the entire sample of 507 goats (Table 3), frequencies of CC homozygotes at *CIITA*_161_C > T (0.98) and TT homozygotes at *CIITA*_481_A > T (0.98) were very high. Frequencies of GG, GG, and AA homozygotes were also relatively high at *ESYT1_559_G > C, HSPA8*_1024_A > G, and *HSPA8*_1064_A > G (0.75, 0.69, and 0.65, respectively). Genotypic frequencies for SNP polymorphisms in *STAT5B, SERPING1,* and *ATP2A3* were more balanced than those of the other genes considered in the study. Frequencies of the C allele at *CIITA*_161_C > T and T allele at *CIITA*_481_A > T were very high (0.99). Frequencies of the G allele at *ESYT1*_559_G > C and *HSPA8*_1024_A > G and the A allele at *HSPA8*_1064_A > G likewise exceeded 0.80, but frequencies of the most common allele at the remaining loci were moderate, ranging from 0.52 to 0.76 (Table 3). When Chinese and Bangladeshi goat breeds were considered as separate populations, similar trends were observed for allelic and genotypic frequencies in all candidate genes (Table 4).

**Table 3:** Genotypic and allelic frequencies for each SNP in total population of goats

SNP	Genotypic frequencies			Allele frequencies	
*CIITA*_161_C > T	CC	CT	TT	C	0.99
	0.98	0.02	0.002	T	0.01
*CIITA*_481_A > T	AA	AT	TT	A	0.01
	0.004	0.02	0.98	T	0.99
*HSPA8*_1024_A > G	AA	AG	GG	A	0.18
	0.04	0.27	0.69	G	0.82
*HSPA8*_1064_A > G	AA	AG	GG	A	0.80
	0.65	0.27	0.04	G	0.20
*ESYT1*_559_G > C	GG	GC	CC	C	0.14
	0.75	0.22	0.03	G	0.86
*STAT5B*_197_A > G	AA	AG	GG	A	0.52
	0.29	0.45	0.26	G	0.48
*SERPING1*_312_C > T	CC	CT	TT	C	0.76
	0.58	0.37	0.05	T	0.24
*SERPING1*_615_G > T	GG	GT	TT	G	0.74
	0.58	0.37	0.05	T	0.24
*ATP2A3*_150_A > G	AA	AG	GG	A	0.27
	0.12	0.31	0.57	G	0.73
*ATP2A3*_680_A > C	AA	AC	CC	A	0.74
	0.58	0.33	0.09	C	0.26

**Table 4:** Genotypic and allelic frequencies and chi-squire tests of HWE genotypic frequencies for each SNP in Chinese and Bangladeshi goats

SNP	Chinese goats						Bangladeshi goats					
	Genotypic frequencies			Allele frequencies		*P*-value for *χ*^2^ test of HWE	Genotypic frequencies			Allele frequencies		*P*-value for *χ*^2^ test of HWE
*CIITA*_161_C > T	CC	TC	TT	C	0.98	0.006	CC	TC	TT	C	1.00	0.0001
	0.97	0.03	0.01	T	0.02		1	0	0	T	0.00	
*CIITA*_481_A > T	AA	AT	TT	A	0.02	0.006	AA	AT	TT	A	0.01	0.0001
	0.01	0.02	0.97	T	0.98		0.004	0..004	0.99	T	0.99	
*HSPA8*_1024_A > G	AA	AG	GG	A	0.19	0.034	AA	AG	GG	A	0.16	0.61
	0.05	0.26	0.69	G	0.81		0.02	0.28	0.70	G	0.84	
*HSPA8*_1064_A > G	AA	AG	GG	A	0.80	0.04	AA	AG	GG	A	0.83	0.17
	0.68	0.27	0.05	G	0.20		0.69	0.29	0.02	G	0.17	
*ESYT1*_559_G > C	CC	GC	GG	C	0.16	0.0003	CC	GC	GG	C	0.12	0.88
	0.05	0.19	0.76	G	0.84		0.02	0.20	0.78	G	0.88	
*STAT5B*_197_A > G	AA	AG	GG	A	0.49	0.03	AA	AG	GG	A	0.56	0.62
	0.27	0.43	0.30	G	0.51		0.32	0.48	0.20	G	0.44	
*SERPING1*_312_C > T	CC	TC	TT	C	0.71	0.68	CC	TC	TT	C	0.83	0.09
	0.50	0.42	0.08	T	0.29		0.66	0.33	0.01	T	0.17	
*SERPING1*_615_G > T	GG	GT	TT	G	0.76	0.66	GG	GT	TT	G	0.84	0.09
	0.52	0.40	0.08	T	0.24		0.67	0.31	0.02	T	0.16	
*ATP2A3*_150_A > G	AA	AG	GG	A	0.38	0.62	AA	AG	GG	A	0.13	0.0001
	0.16	0.44	0.40	G	0.62		0.06	0.15	0.79	G	0.87	
*ATP2A3*_680_A > C	AA	AC	CC	A	0.63	0.45	AA	AC	CC	A	0.89	0.83
	0.40	0.45	0.15	C	0.37		0.80	0.19	0.01	C	0.11	

*HWE* Hardy-Weinberg equilibrium, ***χ***^**2**^ Chi-squire

Chi-square tests revealed that departures of observed genotypic frequencies from HWE expectations were significant for *CIITA*_161_C > T, *CIITA*_481_A > T, *HSPA8*_1024_A > G, *HSPA8*_1064_A > G, *ESYT1_559_*G > C, and *STAT5B*_197_A > G in Chinese goats and *CIITA*_161_C > T, *CIITA*_481_A > T, and *ATP2A3*_150_A > G in Bangladeshi goats (Table 4). Significant departures from HWE conditions for *CIITA*_161_C > T and *CIITA*_481_A > T in both populations are likely spurious results, reflecting very high allelic frequencies for the most common allele and limited sample sizes. Genotypic frequencies at other loci did not differ from HWE expectations

### Population genetic diversity

Indicators of population genetic diversity such as the *He, Ne*, and *PIC* were highest for *STAT5B*_197_A > G and highest for YCW goats (0.50, 2.00, and 0.38, respectively) followed by CCB, BBG, ESB, and NJY goats. The lowest values for *He, Ne*, and *PIC* were observed for *CIITA* in all goat breeds. Values for *He, Ne*, and *PIC w*ere relatively high for *SERPING1* and *ATP2A3* in all goats breeds but were consistently moderate for the remaining loci (Table 5). Based on *PIC*, *STAT5B, SERPING1*, and *ATP2A3* were categorized as having medium genetic diversity (0.25 < *PIC* < 0.50) for all goat breeds. *CIITA, HSPA8*, and *ESYT1* were categorized as having low genetic diversity (*PIC* < 0.25) for all breeds except CCB and NJY for *HSPA8*_1024_A > G and *HSPA8*_1064_A > G and YCW for *ESYT1*_559_G > C, which were classified as having medium genetic diversity (Table 5).

**Table 5:** Heterozygosity (*He*), effective allele number (*Ne*), and polymorphism information content (*PIC*) for each SNP and breed

SNP	Parameter	Goat breeds				
		YCW 32	NJY 56	ESB 37	CCB 155	BBG 227
*CIITA*_161_C > T	*He*	0.03	0.02	0.00	0.06	0.00
	*Ne*	1.03	1.02	1.00	1.06	1.00
	*PIC*	0.03	0.02	0.00	0.06	0.00
*CIITA*_481_A > T	*He*	0.03	0.02	0.05	0.05	0.01
	*Ne*	1.03	1.02	1.05	1.05	1.01
	*PIC*	0.03	0.02	0.05	0.05	0.01
*HSPA8*_1024_A > G	*He*	0.19	0.32	0.10	0.36	0.27
	*Ne*	1.23	1.46	1.11	1.55	1.38
	*PIC*	0.17	0.27	0.10	0.29	0.24
*HSPA8*_1064_A > G	*He*	0.17	0.32	0.10	0.36	0.29
	*Ne*	1.20	1.46	1.12	1.56	1.40
	*PIC*	0.16	0.27	0.1	0.29	0.25
*ESYT1*_559_G > C	*He*	0.34	0.17	0.15	0.30	0.21
	*Ne*	1.52	1.20	1.18	1.44	1.26
	*PIC*	0.28	0.15	0.14	0.26	0.19
*STAT5B*_197_A > G	*He*	0.50	0.48	0.49	0.49	0.49
	*Ne*	2.00	1.92	1.96	2.00	1.97
	*PIC*	0.38	0.36	0.37	0.37	0.37
*SERPING1*_312_C > T	*He*	0.45	0.39	0.41	0.41	0.28
	*Ne*	1.82	1.65	1.68	1.69	1.39
	*PIC*	0.35	0.32	0.32	0.33	0.24
*SERPING1*_615_G > T	*He*	0.45	0.40	0.41	0.41	0.28
	*Ne*	1.82	1.66	1.68	1.69	1.39
	*PIC*	0.35	0.32	0.32	0.33	0.24
*ATP2A3* _150_A > G	*He*	0.44	0.49	0.49	0.46	0.23
	*Ne*	1.79	1.96	1.98	1.86	1.30
	*PIC*	0.34	0.37	0.37	0.36	0.21
*ATP2A3*_680_A > C	*He*	0.44	0.49	0.49	0.45	0.19
	*Ne*	1.79	1.96	1.98	1.83	1.24
	*PIC*	0.34	0.37	0.37	0.35	0.18

Numbers of goats in each breed are shown in parentheses under the breed designation

*YCW* Yichang White, *NJY* Nanjiang Yellow, *ESB* Enshi Black, *CCB* Chinese crossbred, *BBG* Black Bengal Goat

### Population association analysis

Association analysis confirmed that 10 SNP from six genes (*CIITA, HSPA8, STAT5B, ESYT1, ATP2A3*, and *SERPING1*) were significantly associated with FEC, with nominal significance levels of *P* < 0.05 to *P* < 0.01 (Table 6). Highly significant (*P* < 0.01) associations were observed for *HSPA8*_1024_A > G, *HSPA8*_1064_A > G, *SERPING1*_312_C > T, *SERPING1* 615_G > T, and *ATP2A3_*680_A > C (Table 6). Large differences were observed between TT and CC genotypes (*P* = 0.003) and TT and TC genotypes (*P* = 0.004) at *SERPING1*_312_C > T, between TT and GG genotypes (*P* = 0.002) and TT and GT genotypes (*P* = 0.005) at *SERPING1*_615_G > T, and between CC and AA genotypes (*P* = 0.012) at *ATP2A3*_680_A > C. Differences between some others genotypes also reached lower levels of significance (*P* < 0.05) (Table 6).

**Table 6:** Associations between SNP and fecal egg counts

Gene	SNP	Genotype			Association *P*-value	Multiple comparison test *P*-values		
		Number						
*CIITA*	*CIITA*_161_C > T	CC	CT	TT	0.039*	TC-CC	TT-CC	TT-TC
		494	9	1		0.046*	0.828	0.977
	*CIITA*_481_A > T	AA	AT	TT	0.037*	AT-AA	TT-AA	TT-AT
		2	8	495		0.977	0.822	0.043*
*HSPA8*	*HSPA8*_1024_A > G	AA	AG	GG	0.014**	AG-AA	GG-AA	GG-AG
		20	138	348		0.097	0.547	0.031*
	*HSPA8*_1064_A > G	AA	AG	GG	0.014**	AG-AA	GG-AA	GG-AG
		344	138	20		0.031*	0.552	0.098
*ESYT1*	*ESYT1*_559_G > C	GG	GC	CC	0.024*	GC-CC	GG-CC	GG-GC
		381	107	17		0.029*	0.132	0.202
*STAT5B*	*STAT5B*_197_A > G	AA	AG	GG	0.021*	AG-AA	GG-AA	GG-AG
		144	224	127		0.849	0.129	0.023*
*SERPING1*	*SERPING1*_312_C > T	CC	TC	TT	0.003**	TC-CC	TT-CC	TT-TC
		292	189	25		0.998	0.003**	0.004**
	*SERPING1*_615_G > T	GG	GT	TT	0.003**	GT-GG	TT-GG	TT-GT
		291	189	25		0.998	0.002**	0.005**
*ATP2A3*	*ATP2A3*_150_A > G	AA	AG	GG	0.044*	AG-AA	GG-AA	GG-AG
		59	155	290		0.102	0.038*	0.925
	*ATP2A3*_680_A > C	AA	AC	CC	0.009**	AC-AA	CC-AA	CC-AC
		289	167	45		0.886	0.012**	0.037*

* *P* < 0.05, ** *P* < 0.01

Means and standard errors for FEC, PCV, and body weight (BW) for each genotype for candidate genes with significant effects on FEC are presented in Table 7. Across all goat breeds, generally undesirable effects (i.e., higher FEC) were associated with the less-frequent allele at all loci except *STAT5B*_197_A > G. Dominance relationships for these unfavorable alleles varied among loci. Additive gene action was indicated only for *ATP2A3*_680_A > C and, perhaps, *ATP2A3*_150_A > G. Allelic frequencies were similar for the two alleles at *STAT5B*_197_A > G, with an apparent recessive favorable effect of the G allele. Particularly large and apparently dominant unfavorable effects of rare minor alleles were observed for both *CIITA* loci. Significant differences among SNP genotypes in PCV were not observed for any of the loci. Effects of genotype on body weight were detected only for the two *ATP2A3* loci, with approximately additive effects for *ATP2A3*_150_A > G, but apparent dominance of the A allele for *ATP2A3*_680_A > C.

**Table 7:** Least-squares means for FEC, PCV and BW of each SNP genotypes in all goats

SNPs ID	Genotype	*n*	All goats		
			FEC	PCV, %	BW, kg
*CIITA*_161_C > T	CC	494	2.00 ± 0.08^a^	26.26 ± 0.21	14.45 ± 0.97
	TC	9	3.05 ± 0.09^b^	25.83 ± 0.34	15.89 ± 6.84
	TT	1	3.08 ± 0.23^ab^	24.00 ± 0.00	27.00 ± 0.00
*CIITA*_481_A > T	AA	2	3.09 ± 0.14^ab^	24.00 ± 0.38	27.00 ± 0.77
	AT	8	3.06 ± 0.07^b^	25.83 ± 0.25	15.89 ± 0.84
	TT	495	2.00 ± 0.09^a^	26.27 ± 0.21	14.46 ± 0.96
*HSPA8*_1024_A > G	AA	20	2.42 ± 0.15^b^	26.45 ± 0.22	16.87 ± 0.43
	AG	138	1.98 ± 0.11^a^	26.13 ± 0.33	14.13 ± 0.83
	GG	348	2.01 ± 0.08^a^	26.28 ± 0.16	14.50 ± 1.03
*HSPA8*_1064_A > G	AA	344	2.01 ± 0.04^a^	26.30 ± 0.18	14.49 ± 0.06
	AG	138	2.00 ± 0.07^a^	26.13 ± 0.33	15.13 ± 0.82
	GG	20	2.42 ± 0.15^b^	26.45 ± 0.32	16.87 ± 0.43
*ESYT1*_559_G > C	GG	381	1.97 ± 0.09^a^	25.32 ± 0.17	14.41 ± 0.94
	GC	107	2.18 ± 0.12^b^	26.23 ± 0.37	15.40 ± 1.02
	CC	17	2.15 ± 0.15^b^	25.32 ± 0.17	16.41 ± 0.75
*STAT5B*_197_A > G	AA	144	2.00 ± 0.06^b^	26.39 ± 0.15	14.96 ± 0.25
	AG	224	1.92 ± 0.07^a^	26.30 ± 0.17	14.36 ± 0.59
	GG	127	2.24 ± 0.08^c^	26.09 ± 0.28	14.53 ± 0.22
*SERPING1*_312_C > T	CC	292	2.01 ± 0.04^a^	26.37 ± 0.26	14.38 ± 0.14
	TC	189	2.00 ± 0.06^a^	26.15 ± 0.16	14.72 ± 0.70
	TT	25	2.31 ± 0.14^b^	25.66 ± 0.43	14.69 ± 0.96
*SERPING1_*615_G > T	GG	291	2.01 ± 0.04^a^	26.37 ± 0.26	14.36 ± 0.12
	GT	189	2.00 ± 0.06^a^	26.15 ± 0.16	14.72 ± 0.70
	TT	25	2.30 ± 0.14^b^	25.66 ± 0.43	14.70 ± 0.96
*ATP2A3*_150_A > G	AA	59	2.20 ± 0.08^c^	25.47 ± 0.19	16.20 ± 0.03^b^
	AG	155	2.14 ± 0.11^b^	26.52 ± 0.25	15.06 ± 0.94^b^
	GG	290	1.92 ± 0.10^a^	26.30 ± 0.15	13.88 ± 0.91^a^
*ATP2A3*_680_A > C	AA	289	1.93 ± 0.04^a^	26.27 ± 0.16	13.90 ± 0.93^a^
	AC	167	2.12 ± 0.06^b^	26.44 ± 0.14	14.75 ± 0.99^a^
	CC	45	2.25 ± 0.11^c^	25.61 ± 0.54	17.32 ± 0.65^b^

*FEC* fecal egg count, *PCV* packed cell volume, *BW* body weight. FEC were transformed as LFEC = log_10_(FEC + 25) before analysis

^a, b, c^Means within a column and SNP with different superscripts differ (*P* < 0.05)

The FEC for Chinese goat breeds were higher than those of Bangladeshi goats (Table 1, Additional file 1: Table S4). There were significant differences for FEC among all the genotypes within significant SNP loci for both Chinese and Bangladeshi goat breeds except for genotypes within *HSPA8* and *CIITA* in Bangladeshi goats.

### Correlation among measured traits

Correlation coefficients (Table 8) between PCV and FEC were negative and significant in Chinese goats (*r* = − 0.45*; *P* < 0.05), Bangladeshi goats (*r* = − 0.44***; *P* < 0.001) and also in total population of goats (*r* = − 0.47; *P* < 0.001). On the other hand, Correlation coefficient between PCV and BW was non-significant in Chinese goats, Bangladeshi goats and total population of goats. The correlations between BW and FEC was significantly positive (*r* = 0.66*; *P* < 0.05) in Chinese goats, but non-significant and negative (− 0.27) in Bangladeshi goats. In total population of goats, the correlation between BW and FEC was highly significant and positive (*r* = 0.56; *P* < 0.001) (Table 8).

**Table 8:** Correlation coefficients among FEC, BW and PCV for Chinese and Bangladeshi goats

Measurement	All goats		Chinese goats		Bangladeshi goats	
	BW	FEC	BW	FEC	BW	FEC
PCV	−0.40	−0.47**	−0.28	−0.45*	0.31	−0.44***
BW	1	0.56***	1	0.66*	1	−0.27

*FEC* fecal egg count, *PCV* packed cell volume, *BW* body weight

*P < 0.05, ** *P* < 0.01, *** *P* < 0.001

### Linkage disequilibrium (LD) and haplotype association analysis

**Fig. 1:**
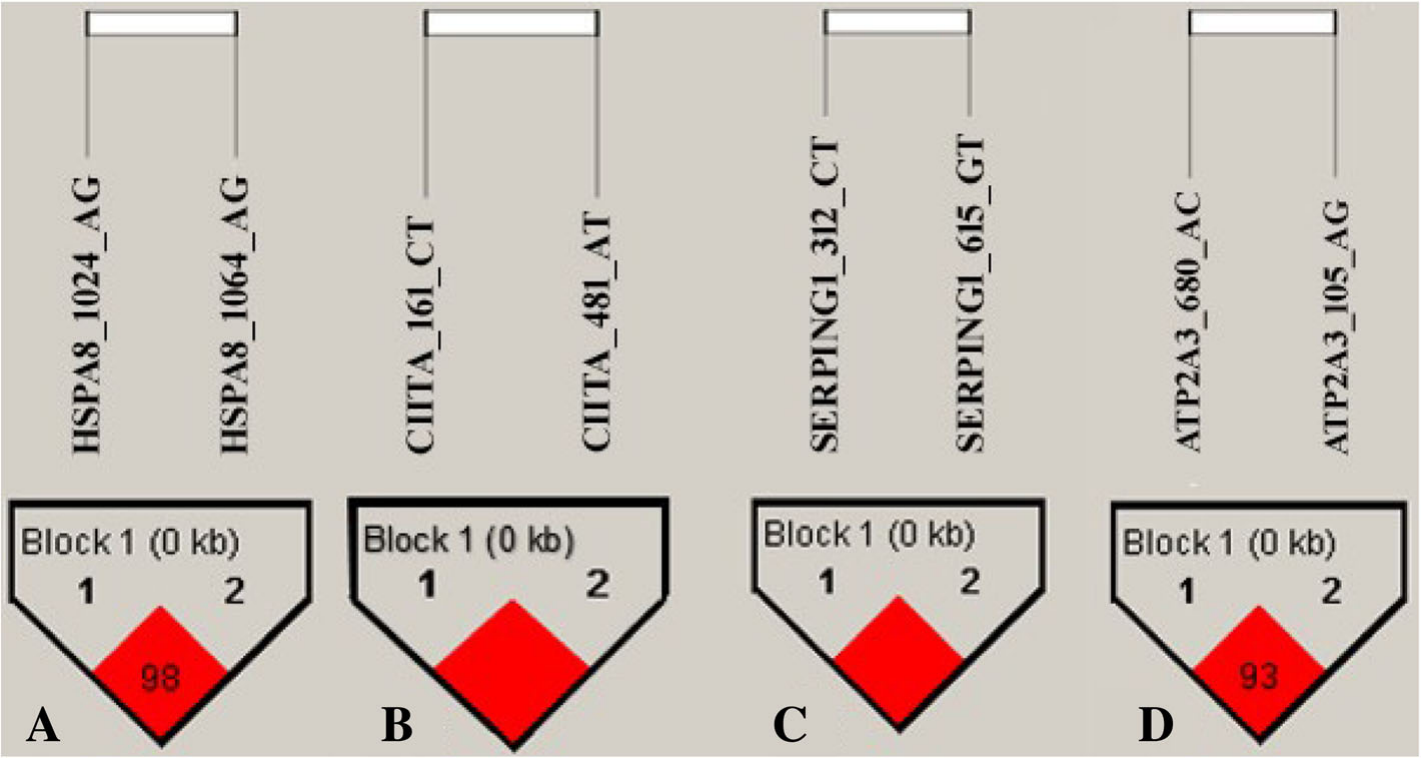
Linkage disequilibrium blocks for SNPs in four genes: **a** *HSPA8*_1024_A > G and *HSPA8*_1064_A > G (LD = 98, *r*^2^ = 0.98); **b** *CIITA*_161_C > T and *CIITA*_481_A > T (LD = 100, *r*^2^ = 1); **c** *SERPING1*_312_C > T and *SERPING1*_615_G > T (LD = 100, *r*^2^ = 1); **d** *ATP2A3*_150_A > G and *ATP2A3*_680_A > C (LD = 93, *r*^2^ = 0.93)

Coefficients of linkage disequilibrium (D´, *r*^2^) were determined for each pair of SNP. Linkage disequilibrium was present between *HSPA8*_1024_A > G and *HSPA8*_1064_A > G, *CIITA*_161_C > T and *CIITA*_481_A > T, *SERPING*1_312_C > T and *SERPING1*_615_G > T, and *ATP2A3_*150_A > G and *ATP2A3*_680_A > C (Fig. 1). In particular, SNP in the intronic region of *SERPING1* and the exon region of *CIITA* were in complete LD (D´ = 100 and *r*^2^ = 1). The SNP variants in the intronic region of *HSPA8* and the 3´ UTR region of *ATP2A3* also expressed strong LD (D´ ≥ 93 and *r*^2^ ≥ 0.93; Fig. 1). High levels of LD between SNP within these genes suggest that significant effects of these SNP in Table 7 were likely associated with common causal regions.

Associations between 13 haplotypes at *CIITA*, *HSPA8*, *SERPING1*, and *ATP2A3* and FEC were examined (Table 9). Haplotypes in *ATP2A3* were associated (*P* < 0.05) with FEC in YCW, NJY, and CCB goats. Associations with FEC (*P* < 0.05) were also observed for haplotypes in *HSPA8* in NJY goats and *SERPING1* in CCB goats. The CCB goats that were heterozygous for the CG and TC chromosome segments had lower FEC compared to those that were homozygous for the TT chromosome segment, but this relationship was not evident for the other breeds of Chinese goats. No significant difference (*P* > 0.05) were found between any of the haplotypes and FEC in BBG goats.

**Table 9:** Associations of haplotype polymorphisms in *CIITA, HSPA8, SERPING1,* and *ATP2A3* with FEC for goat breeds

Gene	Haplotype	YCW		NJY			ESB	CCB		BBG	
		No.	Mean ± SE	No.	(Mean ± SE)	No.	(Mean ± SE)	No.	(Mean ± SE)	No.	(Mean ± SE)
*CIITA*	CCTT	31	2.22 ± 0.12	55	2.06 ± 0.09	37	2.53 ± 0.09		2.44 ± 0.07		1.57 ± 0.03
	TCAT	1	3.15 ± 0.66	1	3.31 ± 0.73	–	–	7	3.19 ± 0.30	–	–
	TTAA	–	–	–	–	–	–	1	3.18 ± 0.79	–	–
*HSPA8*	GGAA	27	2.31 ± 0.13	37	2.09 ± 0.12^b^	32	2.59 ± 0.10	93	2.45 ± 0.08	154	1.58 ± 0.03
	AGAG	4	1.76 ± 0.33	16	1.87 ± 0.18^a^	4	2.13 ± 0.29	50	2.47 ± 0.11	64	1.56 ± 0.05
	AAGG	1	2.51 ± 0.67	3	3.00 ± 0.41^c^		–	11	2.71 ± 0.24	5	1.54 ± 0.17
*SERPING1*	CCGG	14	2.40 ± 0.18	30	2.05 ± 0.13	18	2.48 ± 0.14	77	2.49 ± 0.09^a^	152	1.57 ± 0.03
	TCGT	14	2.13 ± 0.18	20	2.16 ± 0.17	17	2.59 ± 0.14	67	2.37 ± 0.08^a^	71	1.55 ± 0.04
	TTTT	4	2.14 ± 0.34	5	2.07 ± 0.34	2	2.48 ± 0.42	11	2.98 ± 0.24^b^	3	2.09 ± 0.21
*ATP2A3*	AGAC	15	2.13 ± 0.18^b^	20	2.32 ± 0.16^b^	15	2.56 ± 0.15	70	2.50 ± 0.09^a^	32	1.58 ± 0.07
	GGAA	14	2.46 ± 0.18^c^	22	1.83 ± 0.15^a^	13	2.31 ± 0.16	61	2.36 ± 0.10^a^	178	1.59 ± 0.38
	AACC	3	1.89 ± 0.15^a^	14	2.14 ± 0.19^a^	9	2.80 ± 0.19	15	2.89 ± 0.21^b^	3	1.39 ± 0.21
	AAAC	–	–	–	–	–	–	3	2.97 ± 0.46^b^	11	1.39 ± 0.11

Numbers of the digit in bracket indicate the number of the individuals in the group

^a, b, c^ Means within a column and breed with different superscripts differ (*P* < 0.05)

### Artificial challenge trial

**Fig. 2:**
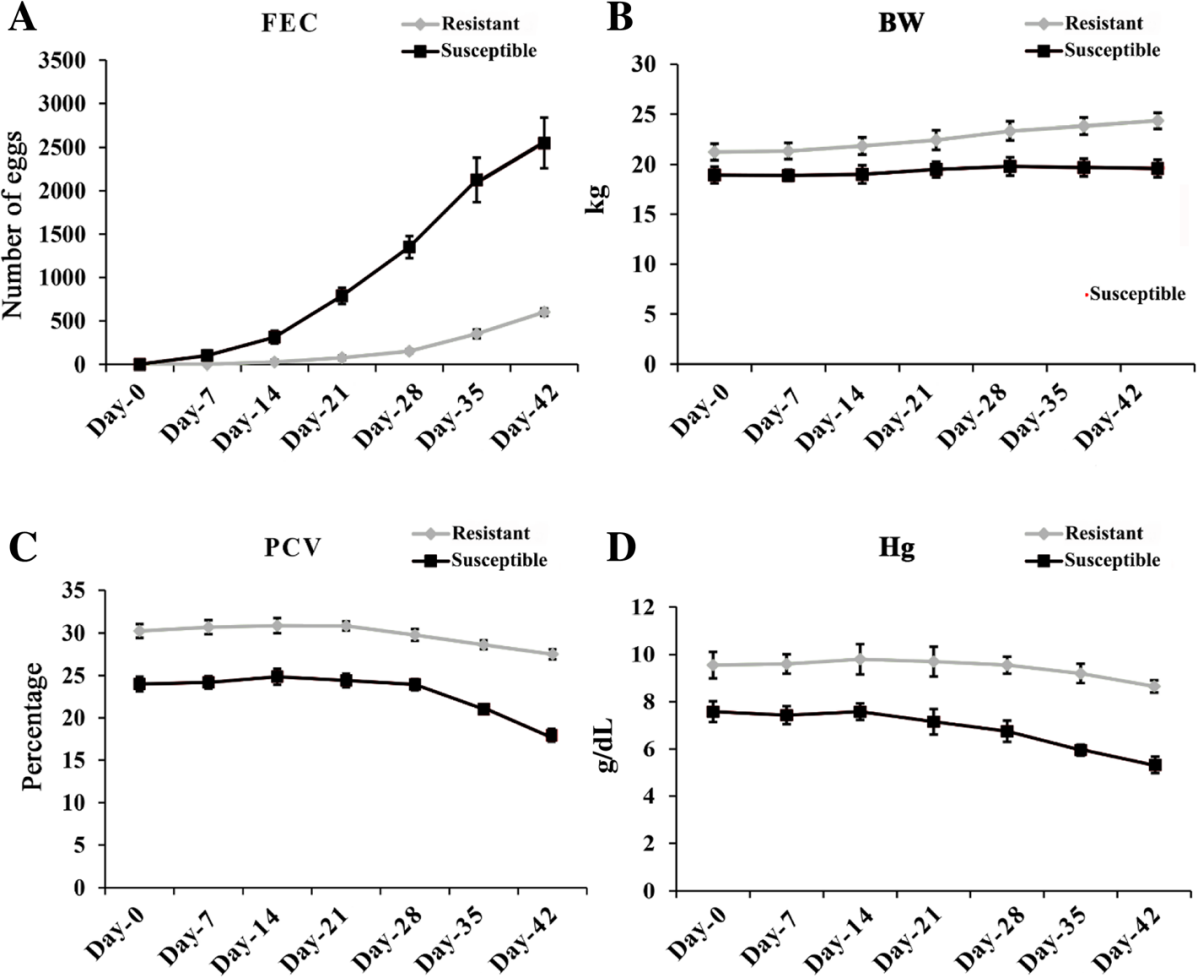
Means for (**a**) fecal egg count, FEC; **b** body weight, BWT; **c** packed cell volume, PCV and **d** haemoglobin (Hg) level in resistant and susceptible groups of Yichang white goats at 0, 7, 14, 21, 28, 35 and 42 days after artificial challenge with 5000 infective L3 larvae of *Haemonchus contortus* cultured under in vitro condition. Day 0 was the day of inoculation with the L3 larvae

The FEC increased gradually from day 21 through days 42 and was higher in susceptible goats compared to resistant goats during that period (Fig. 2a). Additionally, the body weight of resistant goats increased throughout the experimental periods whereas that of susceptible goats was stable during the experimental period (Fig. 2b). PCV and Hg level began to decline at days 28 and 14, respectively, in susceptible goats and continued to decline through day 42 (Fig. 2c and d). On the other hand, PCV and Hg values in resistant goats did not decline as rapidly as those in susceptible goats (Fig. 2c and d).

### Differential gene expression analysis

**Fig. 3:**
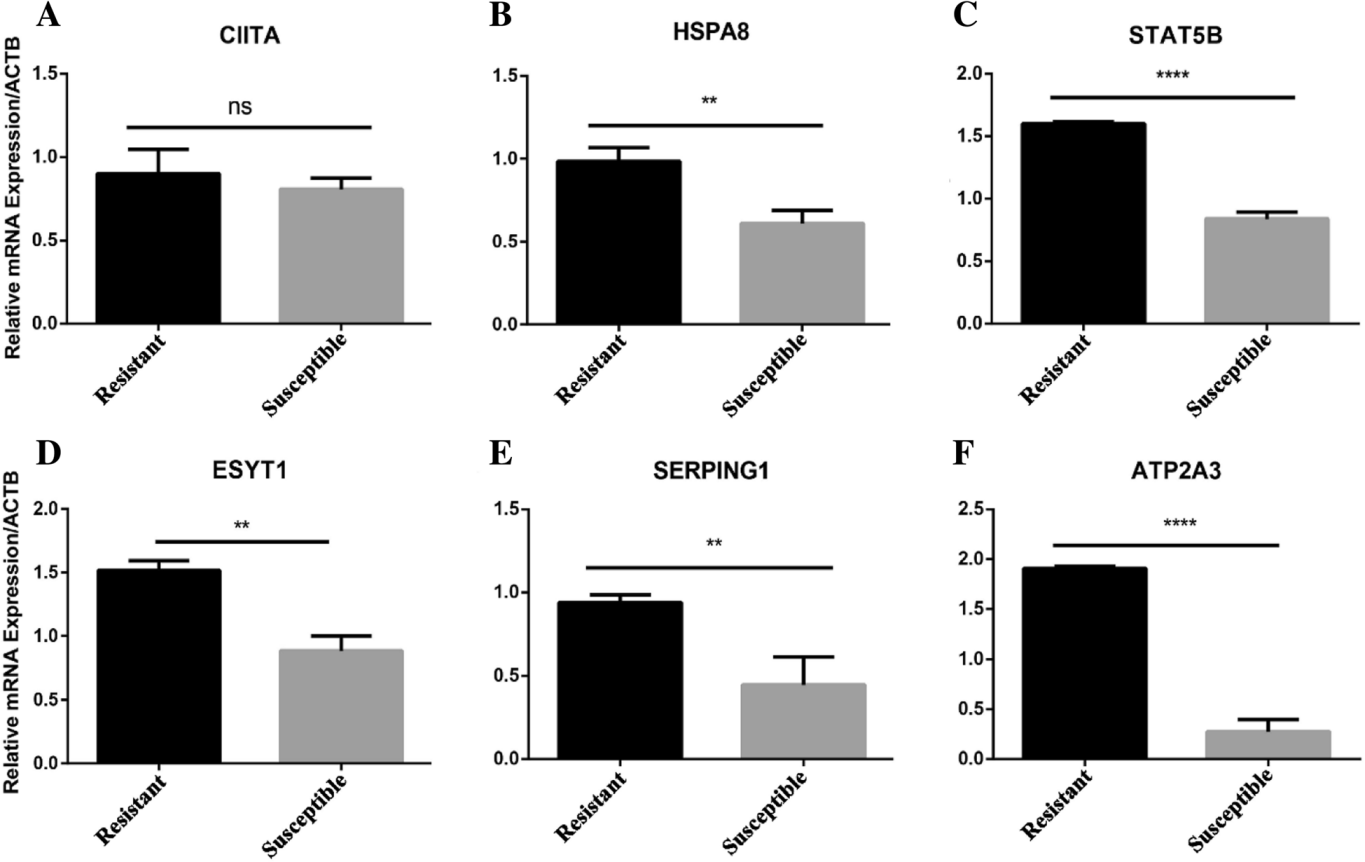
Quantitative reverse-transcriptase polymerase chain reaction (qRT-PCR) measurements of gene expression in abomasal tissue of resistant and susceptible goats. (**a**), (**b**), (**c**), (**d**), (**e**) and (**f**): are the relative mRNA expression of Major histocompatibility complex class II molecules (*CIITA*), heat stress protein 70 (*HSPA8*), signal transducer activator of transcription 5B (*STAT5B*), extended synaptotagmin-1 (*ESYT1*), serpin peptidase inhibitor clade G member 1 (*SERPING1*), and the endoplasmic Ca2+ pump (*ATP2A3*), respectively. Black colour bar represent the resistant group and ash colour bar represent the susceptible group of goats mRNA expression level. Error bars represent standard errors of the means. ** *P* < 0.001, **** *P* < 0.0001 by Student’s *t*-test assuming unequal variances

Relative levels of expression of mRNA in resistant goats were higher than those in susceptible goats for *HSPA8, ESYT1* and *SERPING1* (all *P* < 0.001) and for *ATP2A3* and *STAT5B* (both *P* < 0.0001) (Fig. 3). Resistant and susceptible goats did not differ in expression of *CIITA.*

## Discussion

The FEC is an important phenotypic marker for resistance to *H. contortus* in sheep and goats [42–44]. Host genetics significantly affect FEC and can be used to assess the level of *H. contortus* infection [5]. The current study revealed that Black Bengal goats of Bangladesh had lower FEC than Chinese goat breeds, supporting previous work [4]. Variation among the breeds for FEC may have reflected both genetic and non-genetic differences, because the Bangladeshi and Chinese breeds were evaluated separately in their home countries. However, goats in the two locations were maintained under similar environmental condition, feces were collected at the same period, and FEC were determined using the same method, supporting the hypothesis that the breeds differed genetically in resistance. This is the first time that goats from the two countries have been evaluated for resistance to *H. contortus* using comparable experimental protocols.

Ten novel SNP in *CIITA, ATP2A3, HSPA8, STAT5B, ESYT1*, and *SERPING1* genes were present in both Chinese and Bangladeshi goats and may provide a basis for identification of genetic markers associated with *H. contortus* resistance [45]. *CIITA, ATP2A3, HSPA8, STAT5B, ESYT1*, and *SERPING1* have all been shown to affect regulation of the immune system in humans [46–48], laboratory mice [17], and a few livestock species such as yak [16], chicken [19], and sheep [49]. *CIITA* produces MHC class II proteins that are found on surfaces of several vertebrate immune cells and regulate immune responses to various pathogens. A role for this gene in immune function has been clearly demonstrated in mice and humans but only rarely been reported in lower vertebrates such as fishes [50]. *ATP2A3* encodes SERCA Ca^2+^-ATPase enzymes which play a fundamental role in maintaining intracellular homeostasis by supporting pumping of Ca^2+^ into endoplasmic reticulum of muscle cells. Expression of *ATP2A3* has also been reported to be dramatically reduced in human colon, breast, and lung cancers [48, 51]. Mutations in *SERPING1* cause hereditary angioedema in humans [46]. *HSPA8* has been reported have to anti-tumor effects by inducing chemokine production from tumor cells and activation of chemo-attracted dendritic cells via the *TLR4* pathway in mice [38]. Extended synaptotagmin-1 *(ESYT1)* acts as a Ca^2+^-regulated lipid-transfer protein and membrane-fusion regulator, which facilitates intracellular signaling and expression of immune responses to viruses [52]. Polymorphisms in *STAT5B* have been associated with production and growth traits in chicken and cattle [19, 53]. The only reported association between polymorphisms in these genes and GIN resistance in goats was a positive association involving a polymorphism in *ATP2A3* [54]. However, there are several reports [6, 12, 13] of associations between polymorphism in *DRB-1*, *IGF-1*, *IL2*, *IL13*, and *IFNG* and GIN resistance in goats.

Population genetic parameters can be used to characterize genetic diversity within and among populations. The PIC is an indicator of the extent of polymorphism in a population and the value of PIC has been classified as high, intermediate, and low levels of polymorphism for *PIC* > 0.50, 0.25 < *PIC* < 0.50, and *PIC* < 0.25, respectively [34, 53, 55]. Results of the current study revealed low to intermediate levels of polymorphism for all genes except *CIITA* in Black Bengal goats from Bangladesh. Similar or somewhat higher levels of genetic diversity were observed for Chinese goats compared to Bangladeshi, goats. These results indicated that there was adequate genetic diversity for selection to be effective in controlling GIN infections for all genes except, perhaps, *CIITA*. The SNP polymorphisms in *CIITA* resulted in amino acid substitutions in the gene product and therefore had potential to have a functional association with GIN resistance. Given the low frequency of the deleterious allele at *CIITA*, these polymorphisms may reflect the presence of rare, deleterious mutations. Three significant mutations were detected in the 3′ UTR regions of *STAT5B* and *ATP2A3* and may alter gene regulation and impact gene expression through mechanisms that disrupt miRNA binding [56]. Previous studies have shown that mutations in introns of bovine, caprine, swine and human genes were significantly associated with performance [16, 57–59]. Synonymous mutations can therefore still be used as genetic markers if they are in LD with polymorphisms in functional genes.

Linkage disequilibrium (LD) plays a vital role in mapping genes that affect complex diseases and identifying association among genetic markers and functional genes [60]. Understanding LD among SNP also avoids redundant inferences involving non-independent genetic markers. Result of this study indicated that four pairs of variants were in significant LD with each other. The two polymorphisms in *CIITA* and the two polymorphisms in *SERPING*1 were each in complete LD (*r*^2^ = 1), and, within each gene, represented by only two unique haplotypes. High levels of LD were also observed for polymorphisms in *ATP2A3* (*r*^2^ = 0.93) and *HSPA8* (*r*^2^ = 0.98). Polymorphisms with *r*^2^ > 0.33 are generally considered to be in relatively strong LD [61] and commonly inherited together. When *r*^2^ values are large, haplotype analysis is preferred to the analysis of individual SNP variants [62]. The LD analysis revealed that haplotype differences in *ATP2A3, HSPA8,* and *SERPING1* were significantly associated with FEC.

The artificial challenge trial revealed significantly higher expression levels for all candidate genes except *CIITA* in GIN-resistant, compared to GIN-susceptible, goats. Resistant and susceptible goats were identified by screening a large sample of goats exposed to natural GIN infection. Animals were then dewormed and re-infected with *H. contortus*. Results therefore addressed the consistency of indicators of parasite resistance and subsequent levels of gene expression in different infection cycles. Goats that had lower FEC, indicating greater resistance to infection, under natural grazing conditions appeared much more resistant, based on indicators of parasite resistance, and also had greater expression of five of the six candidate genes in response to a subsequent controlled GIN infection. The high repeatability for indicators of parasite resistance across infection cycles was consistent with results reported for Pelibuey hair sheep [63], and differences in expression of genes involved in immune function have been reported between resistant and susceptible sheep breeds [64]. The results of present study show that the majority of candidate genes selected for this study were differentially expressed in resistant and susceptible goats, further supporting the potential value of SNP in these genes as markers for GIN resistance in goats.

## Conclusions

Population genetic parameter, LD among SNP markers, identification of non-synonymous mutations in candidate genes, differences in relative gene expression between resistant and susceptible goats, and associations with FEC involving both individual SNP genotypes and SNP haplotypes can be used to advance our understanding of options to utilize selective breeding and molecular markers to improve resistance to *H. contortus* and other GIN in goats. Ten SNP within six candidate genes were associated with FEC and provided a suite of potential molecular markers for further study and possible use in screening individuals for resistance to *H. contortus.*

## Additional file

Additional file 1: **Table S1.** Primer sequences and PCR conditions for genotyping SNP in caprine candidate genes. **Table S2.** Cycling condition for Touchdown PCR (TD-PCR) genotyping of SNP. **Table S3.** Primer information for measurement of expression of mRNA by quantitative reverse-transcriptase polymerase chain reaction. **Table S4.** Least-squares means and standard errors for FEC, PCV and BW for each SNP genotype in Chinese and Bangladeshi goats. **Table S5.** Results of association and multiple comparison test *P*-values of non-significant polymorphisms. (DOCX 57 kb)

## Abbreviations

BBG: Black Bengal goats
BW: Body weight
CCB: Crossbred
ESB: Enshi Black
FAM: Fluorescein amidite
FEC: Fecal egg count
GIN: Gastrointestinal nematode
HEX: Hexachloro-Fluorescein
Hg: Hemoglobin
KEGG: Kyoto encyclopedia of genes and genomes
L3: Larval stage 3
LFEC: Logarithmic transformation fecal egg count
NJY: Nanjiang Yellow
ns: non-significant
PCV: Packed cell volume
PIC: Polymorphism information content
SE: Standard error
SNP: Single nucleotide polymorphisms
YCW: Yichang White
