# Supplementary material for: Single nucleotide polymorphisms in candidate genes are significantly associated with resistance to Haemonchus contortus infection in goats
Source: J Anim Sci Biotechnol. 2019 Mar 15;10:30. doi: 10.1186/s40104-019-0327-8 (PMC6419443; doi:10.1186/s40104-019-0327-8)
Supplement: Supplementary file 1 — Table S1. Primer sequences and PCR conditions for genotyping SNP in caprine candidate genes. Table S2. Cycling condition for Touchdown PCR (TD-PCR) genotyping of SNP. Table S3. Primer information for measurement of expression of mRNA by quantitative reverse-transcriptase polymerase chain reaction. Table S4. Least-squares means and standard errors for FEC, PCV and BW for each SNP genotype in Chinese and Bangladeshi goats. Table S5. Results of association and multiple comparison test P-values of non-significant polymorphisms. (DOCX 57 kb) [file 40104_2019_327_MOESM1_ESM.docx]

**Table S1** Primer sequences and PCR conditions for genotyping SNP in caprine candidate genes

| **SNPs ID** | **Allele** | **Forward primer** | **Common primer** | **NCBI Ref.**  **Sequence** |
| --- | --- | --- | --- | --- |
|  |  |  |  |  |
| *CIITA*_161_C>T | X | TCTGGGTCCCCTGCCACC | GAGGTCCCCAGAC  GGTCCAAA | NW_017189511 |
|  | Y | CTTCTGGGTCCCCTGCCACT |  |  |
| *CIITA*_481_A>T | X | CAGAGCCTGAGCAAGGCGGAA | CGAGAAGCCAGCC  ACCTCGAA | NW_017189511 |
|  | Y | CAGAGCCTGAGCAAGGCGGAT |  |  |
| *HSPA8*_1024_A>G | X | CATAAATTCTTTAAATGGAGGA  TTAAGTTTGAT | CCAAGACCTCCAATG  TAACCTAAATGTT | NW_017189501 |
|  | Y | ATAAATTCTTTAAATGGAGGATT  AAGTTTGAC |  |  |
| *HSPA8*_1064_A>G | X | AATCCTCCATTTAAAGAATTTAT  GGTAATAGA | CTTCCTTCTCTGCAGTC  TAAAGAGAAATA | NW_017189501 |
|  | Y | CCTCCATTTAAAGAATTTATGGT  AATAGG |  |  |
| *FAM62A*_559_G>C | X | CCTCCACAAGAAGCCTTTGCG | CTCCCACTTTCCCCAT  CTACTGAAA | NW_017189491 |
|  | Y | CCTCCACAAGAAGCCTTTGCC |  |  |
| *STAT5B*_197_A>G | X | GTGGCTGGATTGAACGGTCCA | TTCCCAGGGAACAAGG  CGACATTTT | NW_017189505 |
|  | Y | GTGGCTGGATTGAACGGTCCG |  |  |
| *SERPING1*_312_C>T | X | CCATCCGCGCCACACACG | CTGGCAGCCCCAGAT  TGAGTCTA | NW_017189501 |
|  | Y | CCCATCCGCGCCACACACA |  |  |
| *SERPING1*_615_G>T | X | CAGAAAACGTAGGGACACGCAC | GTCGGACACGACTGA  GCRACTAAA | NW_017189501 |
|  | Y | AACAGAAAACGTAGGGACACGCAA |  |  |
| *ATP2A3*_150_A>G | X | GGCATGCCCTTAGCAACCCA | TGGTTCCCAGAACTC  CATCAGTGTA | NW_017189505 |
|  | Y | GGCATGCCCTTAGCAACCCG |  |  |
| *ATP2A3*_680_A>C | X | ACACCCTTCTGGCTCATGGGT | GCCSGGTGACCCTTGG  CCTT | NW_017189505 |
|  | Y | CACCCTTCTGGCTCATGGGG |  |  |

**Table S2** Cycling condition for Touchdown PCR (TD-PCR) genotyping of SNP

| **Cycling conditions** |  | **Steps** | **Temperature, °C** | **Time** | **Activity** |
| --- | --- | --- | --- | --- | --- |
| Touchdown (TD)  61-55°C |  | 1 | 94°C | 15 min | Pre-denaturing |
|  |  | 2 | 94°C | 20 Sec | Denaturing |
|  |  | 3 | 61°C | 60 Sec | Touchdown annealing and  Extension, 10 cycles |
|  |  | 4 | Drop - 0.6 / cycle |  |  |
|  |  | Repeat steps 2-3 9 times (a total of 10 cycles) achieving the annealing temperature 55°C | | | |
|  |  | 5 | 94°C | 20 Sec | Denaturing |
|  |  | 6 | 55°C | 60 Sec | Final Annealing and Extension, 26 cycles |
|  |  | Repeat steps 5-6 25 times (a total of 26 cycles) | | | |
| Touchdown (TD)  68-62°C |  | 1 | 94°C | 15 min | Pre-denaturing |
|  |  | 2 | 94°C | 20 Sec | Denaturing |
|  |  | 3 | 68°C | 60 Sec | Touchdown annealing and  Extension, 11 cycles |
|  |  | 4 | Drop - 0.5/ cycle |  |  |
|  |  | Repeat steps 2-3 9 times (a total of 10 cycles) achieving the annealing temperature 55°C | | | |
|  |  | 5 | 94°C | 20 Sec | Denaturing |
|  |  | 6 | 62°C | 60 Sec | Final Annealing and Extension, 26 cycles |
|  |  | Repeat steps 5-6 25 times (a total of 26 cycles) | | | |

**Table S3** Primer information for measurement of expression of mRNA by quantitative reverse-transcriptase polymerase chain reaction

| **Gene** | **Gen Bank accession no.** | **Primer** | **Sequence (5´ - 3´)** | **Product length** |
| --- | --- | --- | --- | --- |
| *ATP2A3* | XM_018064437.1 | Forward | GAGCCCGCCCACAAGTCC | 209 |
|  |  | Reverse | TCCTCTACCGCAGCCACGAT |  |
| *SERPING1* | XM_018059057.1 | Forward | TGACGTCGCCTCCCAGAT | 220 |
|  |  | Reverse | GACAGTCCGGTTAGCCATCG |  |
| *CIITA* | XM_018040957.1 | Forward | CAATTCATCAGGCAACACCAG | 132 |
|  |  | Reverse | GTATCTCTGCTCACCCGGCTT |  |
| *HSPA8* | XM_005689565.3 | Forward | CAGAGTTCAAGCGTAAACACA | 187 |
|  |  | Reverse | AATTCTTCAAACCGGGCAC |  |
| *STAT5B* | NM_001009728.2 | Forward | CCACGACCTGCTTATCAACA | 133 |
|  |  | Reverse | GGTAAAAGGCATCAGATTCCA |  |
| *ESYT1* | XM_005680370.3 | Forward | GACTCCCTCAGGTCACCTCT | 135 |
|  |  | Reverse | CTTGGTGCCCCGGTTCTTAT |  |
| *β- actin* | NM_001101 | Forward | GGACTTCGAGCAGGAGATGG | 233 |
|  |  | Reverse | GCACCGTGTTGGCGTAGAGG |  |

**Table S4** Least-squares means and standard errors for FEC, PCV and BW for each SNP genotype in Chinese and Bangladeshi goats

| **SNP** | **Genotype** | **Chinese goats** | | | | **Bangladeshi goats** | | | |
| --- | --- | --- | --- | --- | --- | --- | --- | --- | --- |
|  |  | **No.** | **LFEC** | **PCV** | **BW** | **No.** | **LFEC** | **PCV** | **BW** |
| *CIITA*_161_C>T | CC | 269 | 2.35±0.16^a^ | 26.04±3.52 | 16.65±6.94 | 225 | 1.58± 0.07 | 26.52±2.78 | 14.84±6.04 |
|  | TC | 9 | 3.20±0.15^b^ | 25.83±2.85 | 15.89±6.94 | - | - | - | - |
|  | TT | 1 | 3.18±0.00^ab^ | 24.00±0.00 | 27.00±0.00 | - | - | - | - |
| *CIITA*_481_A>T | AA | 1 | 3.18±0.00^ab^ | 24.00±0.00 | 27.00±0.00 | 1 | 2.51±0.00 | 27.00±0.00 | 20.00±0.00 |
|  | AT | 7 | 3.20±0.14^b^ | 25.83±2.85 | 15.89±6.83 | 1 | 2.09±0.00 | 21.50±0.00 | 17.00±0.00 |
|  | TT | 271 | 2.35±0.11^a^ | 26.04±3.51 | 16.63±6.93 | 224 | 1.57±0.07 | 26.54±2.78 | 14.85±6.04 |
| *HSPA8*_1024_A>G | AA | 15 | 2.76±0.14^b^ | 26.13±2.99 | 18.8±6.02 | 5 | 1.54±0.28 | 27.40±1.96 | 15.10±3.47 |
|  | AG | 74 | 2.28±0.12^a^ | 25.79±3.60 | 16.27±6.83 | 64 | 1.56±0.14 | 26.52±2.95 | 15.65±5.93 |
|  | GG | 191 | 2.38±0.08^a^ | 26.11±3.48 | 16.62±7.03 | 157 | 1.54±0.11 | 26.50±2.72 | 14.91±6.10 |
| *HSPA8*_1064_A>G | AA | 189 | 2.39±0.14^a^ | 25.12±3.50 | 16.62±7.05 | 155 | 1.58±0.11 | 26.52±2.72 | 15.89±6.14 |
|  | AG | 74 | 2.28±0.12^a^ | 25.79±3.60 | 16.27±6.82 | 64 | 1.56±0.14 | 26.52±2.95 | 14.65±5.93 |
|  | GG | 15 | 2.76±0.14^b^ | 26.13±2.99 | 18.8±6.02 | 5 | 1.54±0.28 | 27.40±1.96 | 16.1±3.47 |
| *ESYT1*_559_G>C | GG | 204 | 2.31±0.07^a^ | 25.02±3.57 | 16.2±6.99 | 177 | 1.57±0.06^b^ | 26.56±2.88 | 14.34±6.27 |
|  | GC | 62 | 2.59±0.11^c^ | 25.75±3.30 | 17.66±6.68 | 45 | 1.63±0.09^b^ | 26.36±2.44 | 13.91±4.56 |
|  | CC | 13 | 2.41±0.11^b^ | 24.96±2.28 | 17.92±6.29 | 4 | 1.40±0.12^a^ | 26.50±1.12 | 16.5±5.77 |
| *STAT5B*_197_A>G | AA | 75 | 2.33±0.21^b^ | 26.17±3.59 | 16.75±7.43 | 69 | 1.57±0.14^a^ | 26.63±2.57 | 13.03±6.51 |
|  | AG | 121 | 2.25±0.16^a^ | 25.10±3.47 | 17.00±6.42 | 103 | 1.53±0.07^a^ | 26.55±2.76 | 14.27±5.31 |
|  | GG | 83 | 2.61±0.13^c^ | 25.82±3.44 | 16.11±7.21 | 44 | 1.65±0.12^b^ | 26.61±2.90 | 14.55±6.24 |
| *SERPING*1_312_C>T | CC | 140 | 2.38±0.18^a^ | 26.15±3.62 | 17.14±7.28 | 152 | 1.58±0.09^a^ | 26.58±2.89 | 14.83±5.96 |
|  | TC | 118 | 2.34±0.14^a^ | 25.97±3.46 | 16.25±6.49 | 71 | 1.55±0.11^a^ | 26.45±2.56 | 15.17±6.25 |
|  | TT | 22 | 2.58±0.12^b^ | 25.57±2.65 | 15.64±6.87 | 3 | 2.09±0.19^b^ | 26.33±2.36 | 15.80±2.01 |
| *SERPING1*_615_G>T | GG | 139 | 2.39±0.17^a^ | 26.16±3.63 | 17.10±7.29 | 152 | 1.58±0.17^a^ | 26.56±2.88 | 14.85±5.94 |
|  | GT | 118 | 2.34±0.14^a^ | 25.97±3.46 | 16.25±6.49 | 71 | 1.55±0.14^a^ | 26.45±2.56 | 15.17±6.25 |
|  | TT | 22 | 2.58±0.12^b^ | 25.57±2.65 | 15.67±6.87 | 3 | 2.09±0.19^b^ | 26.33±2.36 | 15.80±2.01 |
| *ATP2A*3_150_A>G | AA | 45 | 2.59±0.07^c^ | 25.41±3.56 | 18.31±6.60 | 14 | 1.40±0.08^a^ | 25.64±1.49 | 14.43±2.93 |
|  | AG | 122 | 2.43±0.14^b^ | 26.48±3.47 | 16.41±6.96 | 33 | 1.57±0.11^b^ | 26.64±2.25 | 15.06±3.95 |
|  | GG | 110 | 2.26±0.06^a^ | 25.83±3.44 | 16.33±7.00 | 180 | 1.59±0.16^b^ | 26.58±2.92 | 15.38±6.41 |
| *ATP2A3*_680_A>C | AA | 111 | 2.26±0.06^a^ | 25.79±3.44 | 16.35±6.98 | 178 | 1.59±0.28^c^ | 26.57±2.93 | 15.37±6.43 |
|  | AC | 124 | 2.43±0.11^b^ | 26.47±3.41 | 16.42±7.05 | 43 | 1.53±0.24^b^ | 26.35±2.19 | 14.95±3.83 |
|  | CC | 42 | 2.52±0.08^c^ | 25.54±3.66 | 16.42±6.46 | 3 | 1.40±0.09^a^ | 26.67±0.47 | 14.75±0.94 |

^a,b,c^Means within a column and SNP with different superscripts differ (P < 0.05).

**Table S5** Results of association and multiple comparison test *P*-values of non-significant polymorphisms

| **Gene Name** | **SNP ID** | **Gene Frequency** | | | **Multiple comparison test**  ***P*-value** | | | **Association**  **P-value** |
| --- | --- | --- | --- | --- | --- | --- | --- | --- |
|  |  | **(Number)** | | |  |  |  |  |
| *LGP2* | 271_CT | CC | TC | TT | TC-CC | TT-CC | TT-TC | 0.8781 |
|  |  | 126 | 166 | 212 | 0.9762 | 0.9295 | 0.9865 |  |
|  | 874_GT | GG | GT | TT | GT-GG | TT-GG | TT-GT | 0.8768 |
|  |  | 214 | 165 | 124 | 0.9761 | 0.9313 | 0.9862 |  |
| *MBL2_A* | 209_CT | CC | TC | TT | TC-CC | TT-CC | TT-TC | 0.2269 |
|  |  | 418 | 86 | 2 | 0.9265 | 0.2181 | 0.2498 |  |
|  | 546_AG | AA | AG | GG | AG-AA | GG-AA | GG-AG | 0.3633 |
|  |  | 245 | 194 | 67 | 0.4325 | 0.7107 | 0.9972 |  |
|  | 658_AG | AA | AG | GG | AG-AA | GG-AA | GG-AG | 0.4059 |
|  |  | 243 | 195 | 65 | 0.4758 | 0.7273 | 0.9992 |  |
| *NLRP12* | 187_CT | CC | TC | TT | TC-CC | TT-CC | TT-TC | 0.7768 |
|  |  | 83 | 204 | 213 | 0.9890 | 0.9392 | 0.8014 |  |
| *NLRP14* | 125_AG | AA | AG | GG | AG-AA | GG-AA | GG-AG | 0.5224 |
|  |  | 366 | 128 | 12 | 0.4491 | 0.1190 | 0.0524 |  |
|  | 481_AG | AA | AG | GG | AG-AA | GG-AA | GG-AG | 0.4405 |
|  |  | 160 | 245 | 96 | 0.8712 | 0.7115 | 0.4153 |  |
|  | 733_GT | GG | GT | TT | GT-GG | TT-GG | TT-GT | 0.4860 |
|  |  | 18 | 141 | 340 | 0.9828 | 0.8142 | 0.5571 |  |
| *NLRP3* | 636_AG | AA | AG | GG | AG-AA | GG-AA | GG-AG | 0.8743 |
|  |  | 11 | 122 | 371 | 0.9800 | 0.9995 | 0.8764 |  |
|  | 837_AG | AA | AG | GG | AG-AA | GG-AA | GG-AG | 0.9746 |
|  |  | 372 | 119 | 13 | 0.9766 | 0.9998 | 0.9954 |  |
| *NLRP5* | 420_CT | CC | TC | TT | TC-CC | TT--CC | TT-TC | 0.1720 |
|  |  | 23 | 140 | 337 | 0.9490 | 0.5861 | 0.3295 |  |
| *NLRP8* | 495_AG | AA | AG | GG | AG-AA | GG-AA | GG-AG | 0.6163 |
|  |  | 4 | 53 | 449 | 0.9474 | 0.9987 | 0.6084 |  |
| *ATP2A3_I* | 1063_GC | CC | GC | GG | GC-CC | GG-CC | GG-GC | 0.4490 |
|  |  | 4 | 47 | 452 | 0.9379 | 0.7756 | 0.5351 |  |
| *ATP2A3_II* | 92_AG | AA | AG | GG | AG-AA | GG-AA | GG-AG | 0.6036 |
|  |  | 27 | 162 | 317 | 0.9701 | 0.7952 | 0.6822 |  |
|  | 210_CT | CC | TC | TT | TC-CC | TT-CC | TT-TC | 0.4984 |
|  |  | 159 | 219 | 124 | 0.5722 | 0.6305 | 0.9990 |  |
|  | 272_AG | AA | AG | GG | AG-AA | GG-AA | GG-AG | 0.6956 |
|  |  | 30 | 152 | 321 | 0.7080 | 0.8485 | 0.8473 |  |
|  | 475_AC | AA | AC | CC | AC-AA | CC-AA | CC-AC | 0.6047 |
|  |  | 2 | 14 | 489 | 0.9577 | 0.8190 | 0.7074 |  |
| *CLEC4L_A* | 215_CT | CC | TC | TT | TC-CC | TT-CC | TT-TC | 0.9699 |
|  |  | 332 | 141 | 29 | 0.9856 | 0.9842 | 0.9964 |  |
|  | 288_AG | AA | AG | GG | AG-AA | GG-AA | GG-AG | 0.9120 |
|  |  | 29 | 141 | 332 | 0.9967 | 0.9664 | 0.9447 |  |
|  | 151_CT | CC | TC | TT | TC-CC | TT-CC | TT-TC | 0.6069 |
|  |  | 183 | 209 | 110 | 0.8461 | 0.6997 | 0.9341 |  |
| *NLRC4B* | 706_AG | AA | AG | GG | AG-AA | GG-AA | GG-AG | 0.8069 |
|  |  | 3 | 74 | 430 | 0.9062 | 0.8642 | 0.9193 |  |
| *TLR4B* | 422_CT | CC | TC | TT | TC-CC | TT-CC | TT-TC | 0.2834 |
|  |  | 207 | 224 | 72 | 0.9179 | 0.4216 | 0.2696 |  |
| *TLR5B* | 338_GC | CC | GC | GG | GC-CC | GG-CC | GG-GC | 0.7070 |
|  |  | 25 | 171 | 306 | 0.9482 | 0.9987 | 0.7015 |  |
| *TLR5C* | 657_AG | AA | AG | GG | AG-AA | GG-AA | GG-AG | 0.7135 |
|  |  | 308 | 173 | 22 | 0.7073 | 0.9861 | 0.9827 |  |
| *TLR7C* | 634_CT | CC | TC | TT | TC-CC | TT-CC | TT-TC | 0.2367 |
|  |  | 103 | 152 | 247 | 0.2159 | 0.5610 | 0.6365 |  |
| *TLR8C* | 260_CT | CC | TC | TT | TC-CC | TT-CC | TT-TC | 0.1343 |
|  |  | 32 | 113 | 358 | 0.1152 | 0.6979 | 0.0548 |  |
|  | 404_AG | AA | AG | GG | AG-AA | GG-AA | GG-AG | 0.3504 |
|  |  | 359 | 115 | 32 | 0.0555 | 0.6978 | 0.1171 |  |
| *TLR3A* | 180_GC | CC | GC | GG | GC-CC | GG-CC | GG-GC | 0.7506 |
|  |  | 155 | 214 | 135 | 0.7748 | 0.8810 | 0.9893 |  |
| *TLR4A* | 525_GC | CC | GC | GG | GC-CC | GG-CC | GG-GC | 0.3002 |
|  |  | 176 | 230 | 95 | 0.9722 | 0.4317 | 0.3026 |  |
|  | 751_CT | CC | TC | TT | TC-CC | TT-CC | TT-TC | 0.2472 |
|  |  | 215 | 219 | 69 | 0.8149 | 0.4444 | 0.2289 |  |
|  | 860_CT | CC | TC | TT | TC-CC | TT-CC | TT-TC | 0.2779 |
|  |  | 212 | 223 | 70 | 0.9109 | 0.4165 | 0.2644 |  |
| *TLR5A* | 808_AG | AA | AG | GG | AG-AA | GG-AA | GG-AG | 0.7207 |
|  |  | 308 | 171 | 24 | 0.7153 | 0.9986 | 0.9541 |  |
| *TLR8A* | 802_AG | AA | AG | GG | AG-AA | GG-AA | GG-AG | 0.8323 |
|  |  | 25 | 101 | 374 | 0.8257 | 0.8620 | 0.9741 |  |
| *TLR9A* | 646_AG | AA | AG | GG | AG-AA | GG-AA | GG-AG | 0.6706 |
|  |  | 341 | 126 | 29 | 0.7025 | 0.9686 | 0.8029 |  |
| *TLR9B* | 207_AG | AA | AG | GG | AG-AA | GG-AA | GG-AG | 0.6701 |
|  |  | 29 | 125 | 347 | 0.7971 | 0.9648 | 0.7057 |  |
| *TLRC9C* | 88_CT | CC | TC | TT | TC-CC | TT-CC | TT-TC | 0.4988 |
|  |  | 49 | 181 | 276 | 0.5436 | 0.8332 | 0.6780 |  |
|  | 732_AC | AA | AC | CC | AC-AA | CC-AA | CC-AC | 0.6979 |
|  |  | 30 | 128 | 344 | 0.8334 | 0.9795 | 0.7214 |  |
| *TLR3B* | 306_CT | CC | TC | TT | TC-CC | TT-CC | TT-TC | 0.7615 |
|  |  | 133 | 215 | 157 | 0.9970 | 0.8696 | 0.7965 |  |
| *TLR6B* | 180_CT | CC | TC | TT | TC-CC | TT-CC | TT-TC | 0.8211 |
|  |  | 65 | 205 | 225 | 0.8116 | 0.9137 | 0.9460 |  |
| *NLX1A* | 68_GC | CC | GC | GG | GC-CC | GG-CC | GG-GC | 0.9215 |
|  |  | 401 | 95 | 8 | 0.9250 | 0.9960 | 0.9784 |  |
| *TLR10B* | 81_CT | CC | TC | TT | TC-CC | TT-CC | TT-TC | 0.9754 |
|  |  | 312 | 169 | 19 | 0.9911 | 0.9801 | 0.9898 |  |
|  | 569_CT | CC | TC | TT | TC-CC | TT-CC | TT-TC | 0.8137 |
|  |  | 190 | 235 | 79 | 0.8064 | 0.9867 | 0.9489 |  |
| *CLEC10A* | 485_AG | AA | AG | GG | AG-AA | GG-AA | GG-AG | 0.6685 |
|  |  | 166 | 225 | 112 | 0.7386 | 0.9974 | 0.7461 |  |
| *CLEC4kA* | 317_AG | AA | AG | GG | AG-AA | GG-AA | GG-AG | 0.9889 |
|  |  | 331 | 154 | 17 | 0.9918 | 0.9977 | 0.9935 |  |
|  | 630_CT | CC | TC | TT | TC-CC | TT-CC | TT-TC | 0.9652 |
|  |  | 393 | 95 | 17 | 0.9701 | 0.9998 | 0.9961 |  |
| *CLEC4K* | 749_CT | CC | TC | TT | TC-CC | TT-CC | TT-TC | 0.6338 |
|  |  | 207 | 232 | 56 | 0.9556 | 0.6575 | 0.7642 |  |
| *CLEC8A* | 51_AG | AA | AG | GG | AG-AA | GG-AA | GG-AG | 0.1702 |
|  |  | 23 | 112 | 370 | 0.9538 | 0.5681 | 0.3420 |  |
|  | 139_AG | AA | AG | GG | AG-AA | GG-AA | GG-AG | 0.1648 |
|  |  | 23 | 114 | 370 | 0.9560 | 0.5687 | 0.3295 |  |
|  | 356_AG | AA | AG | GG | AG-AA | GG-AA | GG-AG | 0.1630 |
|  |  | 23 | 112 | 367 | 0.9586 | 0.5713 | 0.3303 |  |
|  | 575_AG | CC | GC | GG | AG-AA | GG-AA | GG-AG | 0.4686 |
|  |  | 82 | 216 | 202 | 0.0417 | 0.1407 | 0.7798 |  |
| *MINCLE* | 335_GC | CC | GC | GG | GC-CC | GG-CC | GG-GC | 0.9109 |
|  |  | 85 | 153 | 263 | 0.9914 | 0.9904 | 0.9421 |  |
|  | 492_CT | CC | TC | TT | TC-CC | TT-CC | TT-TC | 0.2087 |
|  |  | 231 | 204 | 71 | 0.2532 | 0.9974 | 0.5536 |  |
| *SFTPA 1* | 899_AG | AA | AG | GG | AG-AA | GG-AA | GG-AG | 0.0350 |
|  |  | 394 | 92 | 18 | 0.0659 | 0.5890 | 0.1332 |  |
| *SFTPD* | 148_AG | AA | AG | GG | AG-AA | GG-AA | GG-AG | 0.4016 |
|  |  | 318 | 162 | 26 | 0.8402 | 0.4152 | 0.5992 |  |
|  | 241_CT | CC | TC | TT | TC-CC | TT-CC | TT-TC | 0.4753 |
|  |  | 28 | 174 | 304 | 0.6781 | 0.4920 | 0.8407 |  |
|  | 701_CT | CC | TC | TT | TC-CC | TT-CC | TT-TC | 0.8159 |
|  |  | 165 | 229 | 112 | 0.8785 | 0.9930 | 0.8470 |  |
| *CLEC4K B* | 522_GT | GG | GT | TT | GT-GG | TT-GG | TT-GT | 0.6196 |
|  |  | 210 | 226 | 57 | 0.9916 | 0.6484 | 0.6942 |  |
|  | 1171_GT | GG | GT | TT | GT-GG | TT-GG | TT-GT | 0.5453 |
|  |  | 94 | 248 | 163 | 0.1215 | 0.9374 | 0.1332 |  |
| *CLEC9A* | 1061_AG | AA | AG | GG | AG-AA | GG-AA | GG-AG | 0.9548 |
|  |  | 56 | 176 | 271 | 0.9556 | 0.9844 | 0.9785 |  |
| *OLA-DRA-1* | 379_AG | AA | AG | GG | AG-AA | GG-AA | GG-AG | 0.7721 |
|  |  | 6 | 40 | 456 | 0.7855 | 0.7539 | 0.9996 |  |
|  | 760_AG | AA | AG | GG | AG-AA | GG-AA | GG-AG | 0.9772 |
|  |  | 79 | 242 | 185 | 0.9903 | 0.9772 | 0.9939 |  |
| *OLA-DYB* | 335_CT | CC | TC | TT | TC-CC | TT-CC | TT-TC | 0.3044 |
|  |  | 46 | 162 | 292 | 0.3321 | 0.3153 | 0.9974 |  |
| *CDH 1* | 869_CT | CC | TC | TT | TC-CC | TT-CC | TT-TC | 0.6832 |
|  |  | 388 | 98 | 14 | 0.6998 | 0.9876 | 0.8892 |  |
| *CDKN 1A* | 185_AG | AA | AG | GG | AG-AA | GG-AA | GG-AG | 0.9359 |
|  |  |  |  |  | 0.9437 | 0.9770 | 0.9641 |  |
| *CXCR 4* | 722_CT | CC | TC | TT | TC-CC | TT-CC | TT-TC | 0.5243 |
|  |  | 14 | 95 | 334 | 0.9136 | 0.9998 | 0.5404 |  |
|  | 1088_AG | AA | AG | GG | AG-AA | GG-AA | GG-AG | 0.8574 |
|  |  | 1 | 34 | 470 | 0.9986 | 0.9989 | 0.8535 |  |
|  | 1175_AG | AA | AG | GG | GG-AG | | | 0.3533 |
|  |  | 0 | 28 | 479 | 0.3611 | | |  |
| *FOS* | 315_CT | CC | TC | TT | TC-CC | TT-CC | TT-TC | 0.8564 |
|  |  | 181 | 221 | 102 | 0.8897 | 0.9977 | 0.8931 |  |
|  | 817_CT | CC | TC | TT | TC-CC | TT-CC | TT-TC | 0.6172 |
|  |  | 211 | 226 | 69 | 0.6997 | 0.7326 | 0.9787 |  |
| *NLRP 7* | 1107_AG | AA | AG | GG | AG-AA | GG-AA | GG-AG | 0.8559 |
|  |  | 1 | 88 | 407 | 0.9954 | 0.9996 | 0.8516 |  |
| *TRAF 4 II* | 264_AT | AA | AT | TT | AT-AA | TT-AA | TT-AT | 0.6289 |
|  |  | 299 | 166 | 40 | 0.7660 | 0.8719 | 0.6664 |  |
| *ITGB7* | 175AC | AA | AC | CC | AC-AA | CC-AA | CC-AC | 0.9428 |
|  |  | 59 | 155 | 288 | 0.9980 | 0.9732 | 0.9724 |  |
| *PTPN6* | 396_CT | CC | TC | TT | TC-CC | TT-CC | TT-TC | 0.2390 |
|  |  | 133 | 258 | 113 | 0.7597 | 0.2313 | 0.4613 |  |
| *IL2RA* | 274_AT | AA | AT | TT | AT-AA | TT-AA | TT-AT | 0.9184 |
|  |  | 115 | 228 | 161 | 0.9282 | 0.9361 | 1.0000 |  |
| *IL2RB* | 231_CT | CC | TC | TT | TC-CC | TT-CC | TT-TC | 0.7509 |
|  |  | 397 | 101 | 4 | 0.7446 | 0.9945 | 0.9638 |  |
| *SLC 11A 2* | 306_GC | CC | GC | GG | GC-CC | GG-CC | GG-GC | 0.5990 |
|  |  | 7 | 73 | 421 | 0.9466 | 0.8228 | 0.7009 |  |
|  | 811_CT | CC | TC | TT | TC-CC | TT-CC | TT-TC | 0.6490 |
|  |  | 24 | 138 | 341 | 0.9986 | 0.8997 | 0.7004 |  |
| *COL6A2* | 783_AG | AA | AG | GG | GG-AG | | | 0.0616 |
|  |  | 0 | 52 | 454 | 0.0695 | | |  |
|  | 834_CT | CC | TC | TT | TC-CC | TT-CC | TT-TC | 0.5576 |
|  |  | 341 | 148 | 11 | 0.9739 | 0.5614 | 0.5308 |  |
| *MASP* | 91_CT | CC | TC | TT | TC-CC | TT-CC | TT-TC | 0.5090 |
|  |  | 82 | 213 | 210 | 0.4836 | 0.6985 | 0.8917 |  |
|  | 141_AT | AA | AT | TT | AT-AA | TT-AA | TT-AT | 0.5427 |
|  |  | 200 | 216 | 90 | 0.8432 | 0.7946 | 0.5213 |  |
| *VASP* | 109_GC | CC | GC | GG | GC-CC | GG-CC | GG-GC | 0.2338 |
|  |  | 35 | 189 | 277 | 0.5945 | 0.2897 | 0.6003 |  |
|  | 406_AG | AA | AG | GG | AG-AA | GG-AA | GG-AG | 0.0603 |
|  |  | 240 | 207 | 56 | 0.4841 | 0.0744 | 0.3259 |  |
| *IL6R* | 259_AG | AA | AG | GG | AG-AA | GG-AA | GG-AG | 0.9740 |
|  |  | 4 | 63 | 439 | 0.9850 | 0.9919 | 0.9819 |  |
| *ITGA5B* | 194_AT | AA | AT | TT | AT-AA | TT-AA | TT-AT | 0.3624 |
|  |  | 60 | 192 | 254 | 0.9471 | 0.8518 | 0.3982 |  |
| *MARS* | 628_AG | AA | AG | GG | AG-AA | GG-AA | GG-AG | 0.8932 |
|  |  | 230 | 197 | 71 | 0.9524 | 0.9739 | 0.9060 |  |
| *PIK3R3* | 193_CT | CC | TC | TT | TC-CC | TT-CC | TT-TC | 0.5860 |
|  |  | 356 | 130 | 18 | 0.5903 | 0.9829 | 0.8367 |  |
| *PRLR Pr 173* | 166_CT | CC | TC | TT | TC-CC | TT-CC | TT-TC | 0.6923 |
|  |  | 119 | 231 | 156 | 0.9012 | 0.9573 | 0.7009 |  |
| *ANKRD Pr 36* | 372_AG | AA | AG | GG | AG-AA | GG-AA | GG-AG | 0.9130 |
|  |  | 106 | 246 | 144 | 0.9684 | 0.9161 | 0.9739 |  |
| *IL 12RB2 Pr 144* | 502_CT | CC | TC | TT | TC-CC | TT-CC | TT-TC | 0.5429 |
|  |  | 41 | 164 | 300 | 0.9816 | 0.7803 | 0.6900 |  |
| *PIK3R1 Pr 153* | 716_AG | AA | AG | GG | AG-AA | GG-AA | GG-AG | 0.4441 |
|  |  | 165 | 225 | 116 | 0.8656 | 0.7594 | 0.4487 |  |
| *FAM130A1 Pr41* | 379_AG | AA | AG | GG | AG-AA | GG-AA | GG-AG | 0.7748 |
|  |  | 15 | 167 | 324 | 0.8046 | 0.7638 | 0.9857 |  |
|  | 546_CT | CC | TC | TT | TC-CC | TT-CC | TT-TC | 0.6521 |
|  |  | 282 | 195 | 28 | 0.9631 | 0.6389 | 0.7249 |  |
| *KLRD1 Pr26* | 51_AT | AA | AT | TT | AT-AA | TT-AA | TT-AT | 0.2843 |
|  |  | 416 | 61 | 22 | 0.9977 | 0.2758 | 0.3495 |  |
|  | 125_AT | AA | AT | TT | AT-AA | TT-AA | TT-AT | 0.2661 |
|  |  | 25 | 111 | 367 | 0.2888 | 0.2669 | 0.9930 |  |
|  | 204_CT | CC | TC | TT | TC-CC | TT-CC | TT-TC | 0.5694 |
|  |  | 61 | 237 | 201 | 0.6944 | 0.9707 | 0.6627 |  |
| *RAVER1_Pr10* | 845_AG | AA | AG | GG | AG-AA | GG-AA | GG-AG | 0.3835 |
|  |  | 386 | 97 | 21 | 0.8997 | 0.4186 | 0.3523 |  |
| *ZBTB39 Pr56* | 280_CT | CC | TC | TT | TC-CC | TT-CC | TT-TC | 0.8982 |
|  |  | 37 | 148 | 321 | 0.9011 | 0.9090 | 0.9967 |  |
| *ZNF641 Pr8* | 243_AG | AA | AG | GG | AG-AA | GG-AA | GG-AG | 0.6113 |
|  |  | 47 | 158 | 299 | 0.7590 | 0.9747 | 0.6709 |  |
|  | 421_CT | CC | TC | TT | TC-CC | TT-CC | TT-TC | 0.6157 |
|  |  | 47 | 157 | 298 | 0.7584 | 0.9736 | 0.6756 |  |
| *NLRC3* | 403_CT | CC | TC | TT | TC-CC | TT-CC | TT-TC | 0.9992 |
|  |  | 428 | 61 | 9 | 0.9992 | 1.0000 | 0.9999 |  |
| *NLRP8* | 767_CT | CC | TC | TT | TC-CC | TT-CC | TT-TC | 0.3748 |
|  |  | 155 | 254 | 95 | 0.7945 | 0.7242 | 0.3535 |  |
| *CLEC4L A* | 53_CT | CC | TC | TT | TC-CC | TT-CC | TT-TC | 0.9283 |
|  |  | 336 | 141 | 29 | 0.9567 | 0.9711 | 0.9965 |  |
| *NLRC4B* | 212_CT | CC | TC | TT | TC-CC | TT-CC | TT-TC | 0.8946 |
|  |  | 360 | 126 | 19 | 0.9645 | 0.9311 | 0.8936 |  |
| *TLR4B* | 618_AG | AA | AG | GG | AG-AA | GG-AA | GG-AG | 0.2845 |
|  |  | 71 | 223 | 211 | 0.2708 | 0.4221 | 0.9153 |  |
| *TLR5C* | 326_CT | CC | TC | TT | TC-CC | TT-CC | TT-TC | 0.7078 |
|  |  | 25 | 173 | 309 | 0.9485 | 0.9988 | 0.7023 |  |
| *TLR3* | 576_CT | CC | TC | TT | TC-CC | TT-CC | TT-TC | 0.7613 |
|  |  | 134 | 214 | 156 | 0.9974 | 0.8674 | 0.7980 |  |
|  | 617_CT | CC | TC | TT | TC-CC | TT-CC | TT-TC | 0.7540 |
|  |  | 155 | 214 | 143 | 0.7991 | 0.8540 | 0.9992 |  |
| *TLR9C* | 393_CT | CC | TC | TT | TC-CC | TT-CC | TT-TC | 0.6958 |
|  |  | 344 | 129 | 30 | 0.7210 | 0.9782 | 0.8306 |  |
|  | 426_CT | CC | TC | TT | TC-CC | TT-CC | TT-TC | 0.6986 |
|  |  | 30 | 129 | 346 | 0.8314 | 0.9779 | 0.7237 |  |
| *NAIP* | 562_CT | CC | TC | TT | TC-CC | TT-CC | TT-TC | 0.6779 |
|  |  | 71 | 168 | 265 | 0.6904 | 0.9118 | 0.8075 |  |
| *OLA-DMB* | 167_AG | AA | AG | GG | AG-AA | GG-AA | GG-AG | 0.5144 |
|  |  | 79 | 175 | 249 | 0.6515 | 0.6585 | 0.9972 |  |
| *CDKN1A* | 82_AG | AA | AG | GG | AG-AA | GG-AA | GG-AG | 0.3462 |
|  |  | 260 | 190 | 53 | 0.4527 | 0.5940 | 0.9769 |  |
| *IL10 Pr133* | 624_CT | CC | TC | TT | TC-CC | TT-CC | TT-TC | 0.4722 |
|  |  | 45 | 163 | 295 | 0.4730 | 0.4998 | 0.9838 |  |
| *NOD2A* | 352_AG | AA | AG | GG | AG-AA | GG-AA | GG-AG | 0.9946 |
|  |  | 4 | 40 | 451 | 0.99546 | 0.99722 | 0.99711 |  |
|  | 1035_CT | CC | TC | TT | TC-CC | TT-CC | TT-TC | 0.9752 |
|  |  | 455 | 42 | 5 | 0.97679 | 0.99844 | 0.99979 |  |
| *NOD2B* | 120_CT | CC | TC | TT | TC-CC | TT-CC | TT-TC | 0.9947 |
|  |  | 453 | 41 | 4 | 0.99732 | 0.99709 | 0.9954 |  |
|  | 688_CT | CC | TC | TT | TC-CC | TT-CC | TT-TC | 0.9835 |
|  |  | 278 | 177 | 46 | 0.98489 | 0.99951 | 0.99138 |  |
